# Exploring Augmented Reality Integration in Diagnostic Imaging: Myth or Reality?

**DOI:** 10.3390/diagnostics14131333

**Published:** 2024-06-23

**Authors:** Andrea Lastrucci, Yannick Wandael, Angelo Barra, Renzo Ricci, Giovanni Maccioni, Antonia Pirrera, Daniele Giansanti

**Affiliations:** 1 Department of Allied Health Professions, Azienda Ospedaliero-Universitaria Careggi, 50134 Florence, Italy; andrea.lastrucci@unifi.it (A.L.); wandaely@aou-careggi.toscana.it (Y.W.); barraa@aou-careggi.toscana.it (A.B.); riccire@aou-careggi.toscana.it (R.R.); 2 Centre TISP, Istituto Superiore di Sanità, 00161 Roma, Italy; gvnnmaccioni@gmail.com (G.M.); antonia.pirrera@iss.it (A.P.)

**Keywords:** imaging, technologies, augmented reality

## Abstract

This study delves into the transformative potential of integrating augmented reality (AR) within imaging technologies, shedding light on this evolving landscape. Through a comprehensive narrative review, this research uncovers a wealth of literature exploring the intersection between AR and medical imaging, highlighting its growing prominence in healthcare. AR’s integration offers a host of potential opportunities to enhance surgical precision, bolster patient engagement, and customize medical interventions. Moreover, when combined with technologies like virtual reality (VR), artificial intelligence (AI), and robotics, AR opens up new avenues for innovation in clinical practice, education, and training. However, amidst these promising prospects lie numerous unanswered questions and areas ripe for exploration. This study emphasizes the need for rigorous research to elucidate the clinical efficacy of AR-integrated interventions, optimize surgical workflows, and address technological challenges. As the healthcare landscape continues to evolve, sustained research efforts are crucial to fully realizing AR’s transformative impact in medical imaging. Systematic reviews on AR in healthcare also overlook regulatory and developmental factors, particularly in regard to medical devices. These include compliance with standards, safety regulations, risk management, clinical validation, and developmental processes. Addressing these aspects will provide a comprehensive understanding of the challenges and opportunities in integrating AR into clinical settings, informing stakeholders about crucial regulatory and developmental considerations for successful implementation. Moreover, navigating the regulatory approval process requires substantial financial resources and expertise, presenting barriers to entry for smaller innovators. Collaboration across disciplines and concerted efforts to overcome barriers will be essential in navigating this frontier and harnessing the potential of AR to revolutionize healthcare delivery.

## 1. Introduction

### 1.1. Augmented Reality: Origins and Definitions

Augmented reality (AR) is an innovative technology that enhances our perception of reality by overlaying digital information onto real-world images captured through devices like smartphones or AR glasses. Defined by various dictionaries, AR involves integrating computer-generated imagery seamlessly with the user’s environment [1,2,3]. This blending of virtual and physical elements enriches the user experience, providing interactive and immersive encounters.

AR distinguishes itself from virtual reality by supplementing rather than replacing reality, leveraging existing environmental elements for deeper engagement [4]. Initially utilized in fields like medicine and the military, AR became publicly accessible in 2009 through software applications like, for example Layar [4,5,6]. Since then, its application has expanded, with the development of smartphone and tablet-based AR apps catering to everyday needs, such as navigation and gaming [7].

Companies like Windows and Sony have also contributed to AR advancements with projects like HoloLens and SmartEyeglass [6,7]. Beyond information retrieval, AR projects extend to real-time translations, sports or cultural event data viewing, and interactive gaming experiences. Ongoing developments include augmented reality contact lenses and retina displays aimed at enhancing vision for visually impaired individuals. Two main types of AR exist, namely mobile device-based and computer-based [8,9], each offering distinct applications.

### 1.2. Formulating the Research Hypothesis: Defining the Study’s Direction

#### 1.2.1. Augmented Reality for Health

Over time, augmented reality (AR) technology has advanced significantly, presenting numerous opportunities in the healthcare sector, spanning from education to telemedicine applications [10,11,12,13,14,15]. In medical education, AR and mixed reality (MR) technologies are increasingly acknowledged for their value, extending beyond surgical exploration [10,11]. Studies indicate that students benefit from augmented reality experiences, leading to enhanced learning outcomes. Additionally, research underscores AR’s potential to transform healthcare practices, including medical communication, real-time telemedicine, and tele-mentoring [12,13]. Particularly noteworthy is its positive impact on medical education quality, especially during the COVID-19 pandemic, where it facilitated remote learning and skill development [14,15].

Today, augmented reality is widely recognized as a valuable asset in medicine, with the potential to improve diagnosis, treatment, and disease management. In medical imaging, AR offers detailed insights by overlaying digital data onto imaging studies, aiding in diagnosis and treatment planning. It also facilitates guidance during interventional imaging procedures, leading to more precise interventions [16]. As technology progresses, AR is expected to play an increasingly vital role in medical imaging, enhancing diagnostic accuracy and therapeutic procedures [17]. Real-time recording and monitoring features during imaging interventions could provide crucial data for clinical decisions, while the integration of wearable AR devices may offer constant access to imaging data without disrupting workflows [17].

Moreover, AR is anticipated to continue revolutionizing medical education and training by providing interactive, three-dimensional visualization of complex imaging data, enabling healthcare professionals to acquire advanced skills more effectively [17]. As health information systems become more interconnected, AR integration into clinical workflows is expected to facilitate data sharing and collaboration among healthcare team members, optimizing the management of complex cases.

#### 1.2.2. Augmented Reality Integration into Diagnostic Imaging

AR holds promise as a transformative technology, particularly in the field of diagnostic imaging, offering clinicians powerful tools to improve patient care and outcomes [18,19,20,21,22].

The global market for augmented reality (AR) and virtual reality (VR) in healthcare was valued at USD 1.57 billion in 2022 and is projected to reach USD 13.74 billion by 2032, growing at a compound annual rate of 24.81% [22]. AR overlays digital data onto the real world, revolutionizing medical treatments and education [22]. Factors driving this growth include the increased demand for cardiovascular surgeries, the rise of surgical robots, and the importance of medical visualization. Despite challenges like the lack of training of medical professionals and data security concerns, AR and VR offer significant opportunities for manufacturers and international companies, with potential applications in remote patient monitoring and exposure therapy. Continued research and technological advancements are essential for overcoming current challenges and fully leveraging the potential of AR and VR in healthcare.

The integration of extended reality (ER) technologies, such as augmented reality (AR) and virtual reality (VR), into the medical imaging field has seen a significant surge in exploration over the past decade. This trend is evident in various facets of medical practice, ranging from diagnostic imaging to medical education and training. A comprehensive analysis of scientific publications has shed light on the multifaceted applications of AR in the realm of diagnostic imaging, focusing primarily on modalities like ultrasound, interventional radiology, and computed tomography (CT) [18]. These studies not only assess the efficacy of ER in enhancing diagnostic accuracy, but also delve into its potential to revolutionize patient positioning and medical education within the realm of diagnostic imaging.

One notable area of focus has been the integration of AR into medical education, offering a more interactive and engaging learning experience, particularly in disciplines such as anatomy and patient positioning. However, alongside the recognition of its educational benefits, questions arise regarding the cost effectiveness of implementing such technologies. Nevertheless, the results of analyzed studies suggest that the incorporation of AR into clinical practice holds promise in expanding diagnostic capabilities, improving procedural accuracy, and enhancing the overall patient experience through increased visualization and understanding of medical conditions [18].

Medical imaging plays a crucial role in diagnosing and treating various medical conditions, transforming the field from X-rays to magnetic resonance imaging (MRI) and computed tomography (CT) scans [19]. AR has emerged as a pivotal technology within this landscape, overlaying digital data onto real-world objects or locations. In the medical field, AR has become indispensable in patient examinations, surgery, therapy, and several other fields such as AR-assisted surgery, image-guided intervention and therapy, patient education and rehabilitation, medical education and training, VR training sessions, and remote patient monitoring [19].

Moreover, as medical imaging technologies continue to advance, particularly with the advent of augmented reality and virtual reality with depth 3-dimensional (D3D) imaging, opportunities arise to address challenges inherent in reviewing large datasets [20]. These advancements not only provide enhanced spatial resolution, but also offer depth perception through binocular vision, thus potentially streamlining diagnosis and improving patient care, especially in fields like breast cancer assessment [20].

In the realm of interventional radiology (IR) training, where imaging is also crucial, the synergistic relationship between AR and conventional training methodologies has been a subject of increasing interest [21]. Despite challenges such as small sample sizes and technological constraints, studies have demonstrated the potential of AR to improve procedural accuracy, reduce training duration, and boost trainee confidence [21].

However, it is evident that further research and technological developments are necessary to fully leverage the capabilities of augmented reality in medical education and clinical practice. Addressing the current limitations and challenges, such as the high cost of implementation and technological constraints, will be crucial in realizing the transformative potential of ER technologies in the medical field.

#### 1.2.3. Augmented Reality Integration into Healthcare: The Contribution of COVID-19

The COVID-19 outbreak had a favorable effect on the development of the healthcare AR and VR industry as it made it simpler to train and convey knowledge to healthcare professionals using augmented reality, in particular, in the field of *diagnostic imaging* [22].

We can highlight several key points regarding the impact of the COVID-19 pandemic on the development of AR applications in healthcare, *most of the themes of which relate to diagnostic imaging*, as follows [23,24,25,26,27,28,29,30,31,32,33,34,35,36,37,38]:

- *Facilitating training and knowledge dissemination:* The COVID-19 outbreak simplified the training of healthcare professionals and the dissemination of knowledge through AR technology. This allowed for efficient skills acquisition and knowledge transfer, particularly in scenarios where traditional in-person training was limited due to the pandemic;
- *Addressing staffing challenges:* AR technology helped address staffing challenges by providing innovative solutions for training and empowering healthcare professionals to effectively manage patient care, even in the face of workforce shortages;
- *Enhancing patient education:* AR technology aided doctors in educating patients about their health issues, providing interactive and immersive experiences to enhance understanding and engagement;
- *Utilizing AR and VR for communication and education:* Virtual and augmented reality played a crucial role in communicating and disseminating knowledge about the COVID-19 disease. These technologies facilitated effective communication between healthcare providers and patients, as well as the general public, contributing to disease management and prevention efforts;
- *Virtual rehabilitation and pain management:* AR technology proved useful for virtual rehabilitation and pain management of infected patients during treatment, offering personalized interventions and immersive experiences to improve patient outcomes;
- *Reducing face-to-face interactions:* AR and VR technologies created a platform that lowered the amount of face-to-face interactions by clinicians with infected patients, thereby minimizing the risk of disease transmission and optimizing infection control measures;
- *Improving surveillance systems:* AR technology contributed to the improvement of the surveillance system of the ongoing situation through live video broadcasting, enabling real-time monitoring and data analysis to inform public health interventions;
- *Exploring new opportunities in healthcare that have never been faced or thought of before* [23].

The compilation of studies [23,24,25,26,27,28,29,30,31,32,33,34,35,36,37,38] presents a rich tapestry of potential applications emerging from current research endeavors.

Within the field of diagnostic innovation, study [23] unveils a groundbreaking development, an AR system leveraging deep neural networks for nasopharyngeal swab sampling. This technology, born out of the pressing need for accurate COVID-19 testing, promises to elevate the precision and efficiency of diagnostic procedures, thereby enhancing healthcare outcomes.

Meanwhile, study [24] shines a light on the transformative role of tele-dermatology and mHealth in bridging gaps in dermatological care amid the pandemic. While not directly tied to diagnostic imaging, the integration of AR applications within telemedicine frameworks facilitates remote dermatological consultations, fostering improved access to care in an era of social distancing.

In cardiovascular medicine, study [25] delves into the convergence of virtual and physical realms, including extended realities, albeit not solely focused on diagnostic imaging. Yet, these advancements likely entail the integration of AR applications for surgical planning, procedural guidance, and educational endeavors, offering innovative solutions to navigate the challenges posed by the pandemic. Furthering the discussion, study [26] illuminates the pivotal role of extended reality (XR) technologies in enhancing surgical assistance and training. Through AR-based XR solutions, remote surgical guidance, training opportunities, and mentorship thrive, ensuring the continuous development of surgical skills and knowledge transfer amidst restrictions on in-person interactions, a boon particularly relevant during and after the pandemic. Transitioning to medical education, study [27] undertakes a systematic review of medical student feedback on surgical education during the pandemic. While not exclusively centered on AR, the adoption of AR-enabled platforms likely underpins remote learning initiatives and clinical skills training, mitigating disruptions induced by the pandemic and fostering a seamless transition to digital learning environments. Innovation extends to skills development as evidenced by study [28], which outlines the creation of a low-cost AR training platform for ultrasound proficiency enhancement. This initiative addresses the demand for remote training solutions during the pandemic, empowering healthcare professionals to refine their diagnostic imaging skills irrespective of geographical constraints. Meanwhile, study [29] introduces a computational framework supporting the treatment of bedsores amidst the COVID-19 outbreak, possibly leveraging AR technologies for wound assessment, treatment planning, and patient education, a testament to the comprehensive approach adopted in patient care during these challenging times. Turning to spine surgery, study [30] forecasts the impact of digital transformation on medical education and rehabilitation. While not exclusively focusing on AR, digital technologies, including AR applications, likely facilitate remote medical education, virtual surgical simulations, and rehabilitation programs, offering invaluable support in a time marked by restrictions on in-person interactions. The diagnostic imaging landscape undergoes a transformative shift with study [31], which introduces COVI3D, an automatic COVID-19 CT image-based classification and visualization platform harnessing AR technologies. This innovation, tailored to the exigencies of the pandemic, promises enhanced visualization and interpretation of CT scans, thereby aiding disease management and treatment planning. In the realm of surgical precision, study [32] delineates the optimization of robotic-assisted radical prostatectomy through augmented reality. AR technologies emerge as indispensable tools for surgical planning, navigation, and intraoperative guidance, fostering enhanced precision and favorable patient outcomes amidst pandemic-induced resource constraints. Study [33] delves into the integration of virtual, augmented, and alternate reality in medical education, offering innovative solutions to address disruptions triggered by the pandemic. Although not solely focused on diagnostic imaging, AR applications likely play a crucial role in facilitating remote learning, simulation-based training, and interactive educational experiences. In spine medicine, study [34] explores the potential of XR technology, encompassing augmented reality and virtual reality, for preoperative planning, intraoperative navigation, and postoperative rehabilitation. These comprehensive solutions offer invaluable support amidst the challenges posed by the pandemic, fostering optimal patient care through innovative technology integration. Looking ahead, study [35] contemplates the near-future outlook of orthopedics post-pandemic, foreseeing accelerated technological advancements, including AR-assisted surgical procedures and tele-rehabilitation. These innovations promise to reshape the delivery of orthopedic care, optimizing patient outcomes in a post-pandemic landscape. Study [36] offers recommendations for staff in interventional radiology to navigate the challenges posed by the pandemic, albeit not directly related to AR. Nonetheless, innovations in IR procedures and technologies, including AR-guided interventions, likely ensure the safety of healthcare workers and patients amidst evolving healthcare landscapes. Meanwhile, study [37] delves into the realm of trustworthy and intelligent COVID-19 diagnostic Internet of Medical Things (IoMT) through XR technologies. This pioneering initiative, integrating AR and virtual reality (VR) into diagnostic processes, offers intelligent solutions for COVID-19 detection, monitoring, and management, heralding a new era in healthcare delivery and outcomes optimization. Lastly, study [38] introduces intelligent intraoperative haptic AR navigation for COVID-19 lung biopsy, leveraging AR for real-time navigation and guidance. This innovation holds immense promise in enhancing procedural accuracy and reducing complications, particularly critical in the context of COVID-19 patients, where precise diagnosis and treatment are paramount. In summation, the amalgamation of these studies underscores the transformative potential of AR technologies across various facets of healthcare, with particular focus on imaging, spanning medical education, surgical interventions, diagnostic imaging, and patient care, all of which have been accelerated and enhanced in response to the challenges presented by the COVID-19 pandemic.

### 1.3. The Rationale and the Purpose of This Study

Based on the introductory discourse, leading to the formulation of hypotheses, several crucial questions emerge regarding the integration of AR in medical imaging, as follows:

1. *What do scholars mean by augmented reality in imaging applications and how do they perceive it?*
2. *How has the integration of augmented reality in diagnostic imaging evolved over time?*
3. *What significant advancements and challenges have shaped this intersection and what are the emerging themes/patterns?*
4. *How has augmented reality been integrated in the imaging domain with other innovative technologies (e.g., robotics, artificial intelligence, and augmented reality)?*
5. *In what ways has augmented reality demonstrated its potential to enhance diagnostic capabilities, improve medical training, or transform patient care within the field of radiology, and what are the current obstacles to overcome?*

Systematic reviews serve as a cornerstone for mapping out this terrain, acting as indicators of the consolidation of emerging themes and shedding light on areas demanding further scholarly attention. Therefore, the objective of this study is not to find the best methods but to conduct an *umbrella review* of the systematic reviews published thus far in this domain, with the aim of addressing these pivotal questions. This overview, rooted in a narrative synthesis of systematic reviews, holds immense importance in consolidating evidence, unveiling emerging themes, discerning patterns, and identifying research gaps within the realm of AR integration in medical imaging. Such an approach facilitates a comprehensive understanding of the research landscape, explores heterogeneity across studies and, ultimately, guides future research directions in this rapidly evolving field.

## 2. Methods

In this narrative review, the Academy of Nutrition and Dietetics’ (ANDJ) narrative checklist, specifically designed for narrative reviews, was employed to ensure a structured and transparent review process [39]. This checklist relies on a methodological framework based on key criteria essential for comprehensive analysis and evaluation.

The research focused on the PubMed, Google Scholar, and Scopus databases, with a strong emphasis on the intricate field of imaging technologies. Initially, a preselection phase concentrated on studies deeply rooted in the world of imaging and augmented reality (AR).

Subsequently, a selection methodology based on a qualification algorithm using predefined parameters was adopted [40]. These parameters included:

- The clarity of the study’s rationale as articulated in the introduction;
- An examination of the study design;
- Transparency in describing the methods employed;
- The clarity of the results;
- The alignment of the conclusions with the results;
- Finally, an examination of any potential conflicts of interest.

Each of the first five parameters was rated on a scale from 1 to 5, with a threshold of 3 required for inclusion.

The sixth parameter was assessed in a binary manner (yes/no) to determine the inclusion of the study.

**Figure 1:**
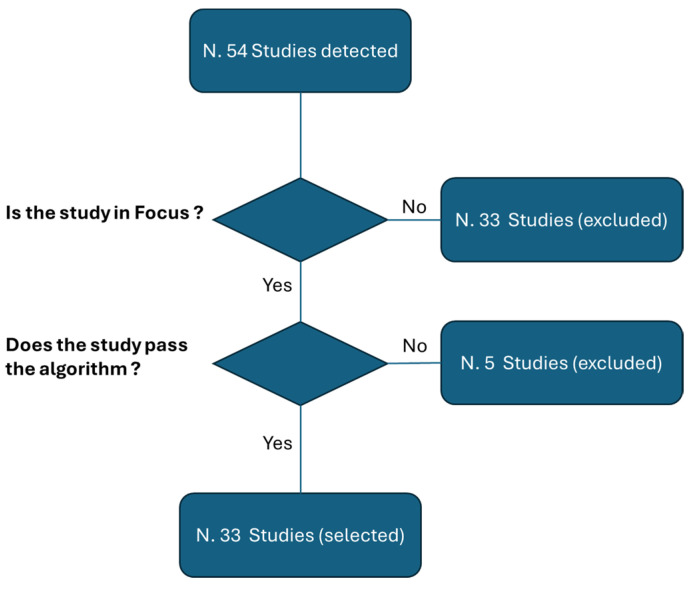
Study selection process.

Only peer-reviewed studies were included in this scholarly examination. The search strategy integrated an AND Boolean operation, weaving together keywords such as “augmented reality”, “AR”, “imaging”, “images”, “image”, and “diagnostic image”. Figure 1 shows the two consequential steps, consisting of a preliminary process of screening and the consequential application of the proposed algorithm. It is, however, important to emphasize that the umbrella review we have proposed is a narrative review.

Our exploration encompassed an analysis of both the “title/abstract” and the “full text” of relevant studies. At the end of the two selection stages, starting from 59 initial studies, 16 systematic reviews were selected [41,42,43,44,45,46,47,48,49,50,51,52,53,54,55,56] (Figure 1).

In addition to the comprehensive search strategy outlined previously, the quest expanded its focus to include the pivotal keyword “COVID-19”. This addition ensured that the literature selected for *constructing the hypotheses* in the introduction and *complementing the discussion* was enriched with insights relevant to the unprecedented challenges posed by the global pandemic. Moreover, the panoramic view of scholarly productions extended beyond traditional databases, encompassing an exploration of prominent national and international institutional websites. Among these domains of medical knowledge, the websites of entities such as the Food and Drug Administration (FDA), the European Union (EU), Health Canada, and the National Health Service (NHS) stood as sources of authoritative information.

The inclusion of these institutional websites enriched the overview with insights from diverse perspectives, providing a comprehensive understanding of the implications of augmented reality in the context of healthcare, particularly amidst the challenges brought forth by the COVID-19 pandemic. This holistic approach ensured that the narrative review was fortified with scholarly discourse and authoritative guidance from both academic and institutional sources.

## 3. Results: Outcome from the Umbrella Review

Below is a comprehensive examination of the studies conducted in this field, organized as follows:

*Trends analysis:* An overview of the trends in this field is presented in Section 3.1. This section provides insights into the evolving patterns and focus of the studies over time.

*Detailed analysis:* Section 3.2 provides comprehensive analysis of each study, both individually and in comparison with one another. This section not only examines the unique contributions and findings of each study, but also categorizes them to highlight common themes and differences. Through this dual approach, the analysis aims to also offer an understanding of the studies, facilitating a deeper insight into their collective impact and significance in the field. This section is supported by two tables (Table 1 and Table 2), which provide detailed data on the studies and the findings.

### 3.1. The Trends in the Studies on Augmented Reality Applications in Imaging

**Figure 2:**
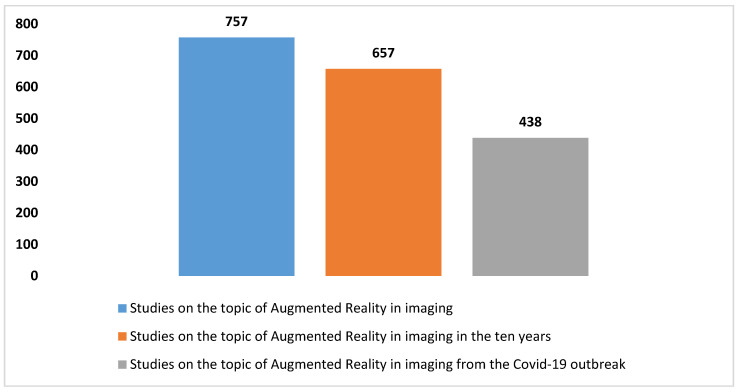
Studies focused on the application of augmented reality in the imaging field.

**Figure 3:**
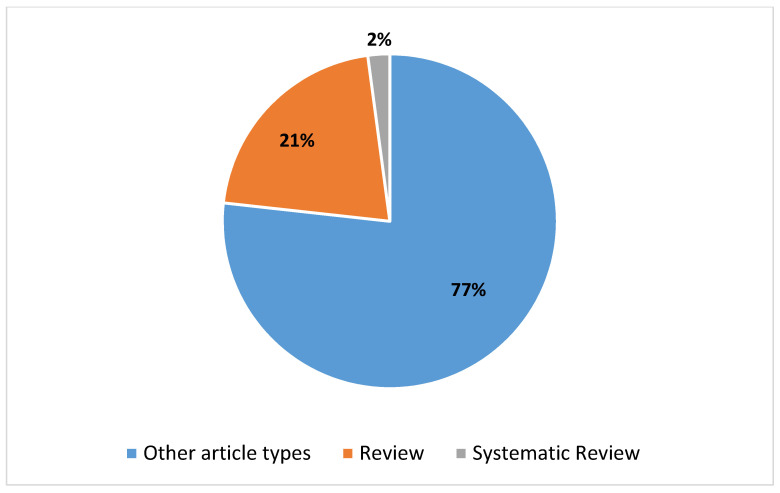
Type of articles focused on augmented reality in the imaging field.

**Figure 4:**
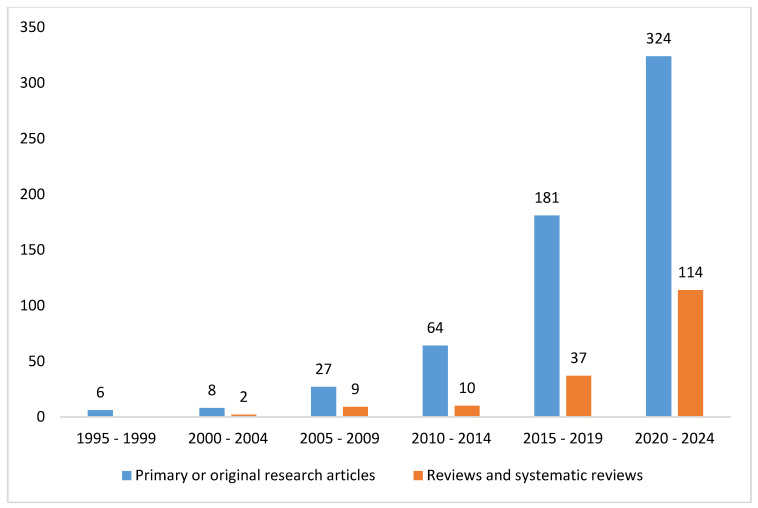
Evolution of augmented reality applications in imaging: a five-year PubMed trend analysis of primary or original research articles and reviews or systematic reviews.

A thorough search was conducted on the PubMed database using specific criteria, yielding a total of 757 studies on the utilization of augmented reality (AR) in imaging from 1995 to the present, as delineated in the composite key in Box 1. The escalating number of articles discovered in PubMed concerning the application of AR in imaging, based on the given search parameters, is illustrated in Figure 2. Additionally, Figure 3 provides insight into the distribution of article types, emphasizing the predominance of reviews (*n* = 18) and systematic reviews (*n* = 3) pertinent to the application of AR in the imaging field. Augmented reality’s role in imaging research has experienced notable accelerations in two distinct time frames, as illustrated in Figure 2. The initial surge occurred over the past decade, with an overwhelming majority (86.8% of articles) of the literature published on PubMed, indicating a substantial uptick in interest in AR’s application in imaging during this period. Subsequently, a further surge in scientific output on this subject is not defined by a specific timeframe, but rather coincides with the advent of the COVID-19 pandemic. Since 2020, a significant proportion, namely 57.9%, of research articles have been published, predominantly propelled by the pandemic’s highlighting of the topic’s relevance and urgency, leading to increased research activity. This reporting period not only illustrates the academic and research communities’ swift response to the challenges posed by COVID-19, but also underscores their resilience and capacity for innovation in times of crisis. The surge in PubMed publications addressing AR applications in imaging underscores significant technological advancements and growing recognition of AR’s capacity to enhance imaging protocols. The remarkable increase in scientific publications on the use of AR in imaging during the COVID-19 pandemic has undoubtedly been facilitated by the technological advancements over the past decade. Figure 3 further elucidates the distribution of article types, particularly emphasizing the prevalence of reviews (*n* = 160) compared to systematic reviews (*n* = 16) pertaining to AR applications in imaging. Figure 4 corroborates the profound interest researchers have recently demonstrated in this domain, offering comprehensive analysis over a five-year period. It delineates the trend of primary or original research articles vis-à-vis reviews or systematic reviews on PubMed concerning this topic. The analysis of Figure 4 underscores that over 85% of all reviews and systematic reviews (*n* = 151 from 2015 to 2024) were published in the last decade, signifying researchers’ focused interest in synthesizing secondary literature aimed at consolidating the vast array of evidence available on the application of AR in imaging. In summary, the increasing body of research on AR’s application in imaging, particularly evident during the COVID-19 pandemic, underscores the pivotal and innovative contributions of AR in this field. This trend not only reflects the resilience and innovative capacity of the field in response to emerging challenges, but also underscores the growing recognition of AR as a pivotal tool for enhancing and refining imaging procedures.

Box 1. **The proposed composite keys.**

*(augmented reality [Title/Abstract]) AND ((imaging [Title/Abstract]))*

*(augmented reality [Title/Abstract])*

### 3.2. Outcome from the Umbrella Review of the Systematic Reviews

The analysis of the results is divided into subsections. Section 3.2.1 reports excerpts from the analyzed systematic review studies with a focus on the contribution of AR in imaging. Section 3.2.1 provides qualitative analysis of the considered systematic reviews according to a defined grid. Section 3.2.2 presents a comparative analysis based on the systematic reviews and emerging themes. In the Supplementary Material, we reported excerpts from the analyzed systematic review studies with a focus on the contribution of AR in imaging for interested scholars.

#### 3.2.1. Qualitative Analysis of the Systematic Reviews

Each systematic review focuses on a specific and targeted theme regarding the integration of AR into imaging, often in conjunction with other emerging technologies. By conducting a qualitative analysis of the *key insights* provided by these reviews, we can effectively highlight the emerging trends and contributions of each study within this domain. This approach allows for a comprehensive examination of the diverse applications and potential impact of AR across various medical specialties and technological contexts.

##### Common Message and Focus on AR and Diagnostic Imaging

Table 1 serves as a comprehensive analysis platform, examining the intricate realms of both imaging and AR. It delves into these two distinct focal points (the true aim of the study), shedding light on their complexities, as portrayed by the findings of each individual study. Through this approach, Table 1 dissects the multifaceted landscape of imaging and AR with two separate focuses, providing a robust foundation for understanding their respective roles and implications.

From the comprehensive examination of the systematic reviews [41,42,43,44,45,46,47,48,49,50,51,52,53,54,55,56], a resounding narrative emerges, underscoring the pervasive utilization and transformative influence of augmented reality AR and VR technologies across diverse surgical domains, spanning orthopedics, neurosurgery, and oncology. Central to this narrative is the profound enhancement in surgical precision, a recurrent theme echoed in systematic reviews authored by Sun et al. [41], Guha et al. [43], Dubron et al. [44], and Sparwasser et al. [49]. These reviews delineate the instrumental role played by AR and VR in refining surgical precision, elucidating its manifold benefits.

Indeed, the systematic reviews elucidate a multifaceted impact of AR, encompassing preoperative simulations, intraoperative guidance, and postoperative rehabilitation, as outlined by Sun et al. [41], Guha et al. [43], Dubron et al. [44], and Sparwasser et al. [49]. By harnessing AR’s capabilities, surgeons can navigate complex anatomical structures with unparalleled precision, thereby mitigating errors and enhancing patient outcomes.

Moreover, the educational landscape within healthcare is undergoing a paradigm shift, sustained by the innovative integration of AR technologies, as underscored by systematic reviews conducted by Kanschik et al. [42], Chidambaram et al. [47], and Ong et al. [50]. These reviews illuminate AR’s transformative potential in immersive learning experiences, fostering the development of surgical competency and technical skills among healthcare professionals.

Furthermore, systematic reviews authored by Seetohul et al. [45], Umana et al. [51], and Rodriguez Peñaranda et al. [53] delve into the profound implications of AR on clinical outcomes and safety. By leveraging advanced technologies like AI, AR, and VR, clinicians can optimize surgical safety, accuracy, and efficiency, thereby elevating patient care standards and reducing complication rates.

However, amidst the optimism surrounding AR’s integration into clinical practice, systematic reviews by Doughty et al. [52], Rodriguez Peñaranda et al. [53], and Unberath et al. [56] shed light on the inherent challenges and prospects that necessitate further exploration by researchers. Technical limitations, data security concerns, accessibility issues, and ethical considerations emerge as focal points warranting meticulous investigation to facilitate the seamless integration of AR into surgical practices.

In summation, the collective insights gleaned from systematic reviews underscore the burgeoning interest among researchers in harnessing AR’s potential within the imaging landscape. AR, VR, and AI technologies hold the promise of reshaping various facets of surgical care, ranging from preoperative planning to intraoperative guidance and postoperative recovery. Nonetheless, concerted research and development endeavors are imperative to surmount the existing challenges and ensure the widespread acceptance and effective implementation of these transformative technologies in daily clinical surgical procedures.

##### Qualitative Analysis in Details

Below is qualitative analysis of each study with key insights.

Sun et al. [41] delve into the transformative potential of VR and AR technology in hip surgery, focusing on preoperative simulation, intraoperative navigation, and postoperative rehabilitation. An analysis of 40 studies reveals that VR and AR can significantly reduce complications, enhance surgical success, and improve precision and safety. The article also explores future AR applications in the operating room and postoperative rehabilitation, indicating the potential for major advancements in surgical practices.

The key insights are as follows:

- Enhanced surgical preparation: VR and AR provide detailed preoperative simulations, leading to better preparedness and fewer intraoperative surprises;
- Real-time navigation: AR improves real-time guidance during surgery, enhancing accuracy and safety;
- Innovative rehabilitation: Postoperative use of VR and AR offers new methods for patient recovery, potentially speeding up rehabilitation and improving outcomes;
- Future prospects: The review suggests that AR can be integrated across different surgical stages to improve patient outcomes and overall surgical efficacy;
- The need for further research: The review emphasizes the necessity for additional comparative studies to evaluate the clinical outcomes and cost effectiveness comprehensively.

Kanschik et al. [42] assess VR and AR applications in the ICU setting, finding a growing interest, but a lack of comprehensive studies. Reviewing 59 studies, the authors note that VR and AR are beneficial for training ICU staff and aiding patients by reducing stress, pain, and improving rehabilitation and communication with relatives.

The key insights are as follows:

- Advanced training tools: VR and AR provide sophisticated training environments for ICU staff, allowing them to practice complex procedures without risks;
- Patient care: These technologies offer immersive experiences that reduce patient anxiety and pain, promoting better mental health and faster recovery;
- Rehabilitation: VR and AR present engaging and effective methods for patient rehabilitation;
- Growing adoption: The review anticipates an increase in the use of VR and AR in ICU settings due to their potential to improve both patient care and staff training.

Focusing on neurosurgery, Guha et al. [43] examine AR for image-guided surgery, detailing its historical development and current applications. Analyzing 33 articles, the review identifies challenges like registration errors, depth perception issues, and temporal asynchrony, but highlights the promise of precise 3D data overlays.

The key insights are as follows:

- Precision in surgery: AR provides accurate overlays of anatomical information, enhancing the precision of neurosurgical procedures;
- Technological challenges: The current limitations include registration errors and synchronization issues between visual and tactile feedback;
- Future improvements: Advances in imaging, registration accuracy, and robotic integration could significantly enhance AR’s utility in neurosurgery.

Dubron et al. [44] explore the use of extended reality (ER), AR, mixed reality (MR), and VR in preoperative planning for orbital fractures. Based on an extensive database search, the review highlights AR’s ability to improve surgical accuracy and precision, especially in incision making and anatomical structure identification.

The key insights are as follows:

- Preoperative planning: AR enhances the accuracy of surgical incisions and the identification of deep anatomical structures;
- Educational benefits: VR offers superior visualization of craniofacial trauma, benefiting surgeon training and education;
- Technological advancements: AR supports precise orientation and fixation of reconstruction plates and patient-specific implants;
- Accuracy margin: A technical accuracy margin of 2–3 mm must be considered for AR applications.

Seetohul et al. [45] discuss the integration of AR into surgical robotic systems, emphasizing the need for improved dexterity and access in minimally invasive surgery. The review evaluates AR’s integration with features like haptic feedback to enhance surgical accuracy.

The key insights are as follows:

- Enhanced robotics: Combining AR with robotic systems improves precision, especially in challenging areas, such as tool placement and depth perception;
- Imaging integration: Advanced imaging and deep learning algorithms play crucial roles in overcoming surgical obstacles;
- Technological synergy: The review highlights the importance of integrating AR with advanced control features to enhance surgical outcomes.

Rodler et al. [46] investigate new imaging technologies in robotic-assisted radical prostatectomy (RARP) for prostate cancer, categorizing 46 studies into primary tumor imaging, intraoperative lymph node detection, and surgeon training.

The key insights are as follows:

- Imaging enhancements: Combining MRI, PSMA-PET CT, and other modalities with RARP improves precision and outcomes;
- Training advances: Advanced imaging technologies enhance surgeon training, improving preparation and skill development;
- Prospective studies: The review underscores the need for prospective studies to confirm the benefits of these combined approaches.

Chidambaram et al. [47] analyze AR’s integration into clinical practice for neurosurgery, focusing on intraoperative visualization. Including 54 studies, it highlights AR’s potential to outperform traditional neuronavigation systems in terms of precision and guidance.

The key insights are as follows:

- Intraoperative visualization: AR offers advanced tools that improve the accuracy and guidance of neurosurgical procedures;
- Technological challenges: Issues like MRI brain segmentation, brain shifts, and coregistration errors need addressing for effective AR use;
- Future integration: Combining AR with artificial intelligence and multimodal imaging could further enhance neurosurgical outcomes.

Bosc et al. [48] categorize AR applications in maxillofacial surgery, analyzing 13 studies. The review emphasizes AR’s accuracy and explores various approaches like those utilizing smart glasses and semi-transparent screens.

The key insights are as follows:

- Surgical applications: AR applications include heads-up guided surgery, semi-transparent screens, direct image projection, and data transfer to monitors;
- High precision: AR provides high accuracy in maxillofacial surgery with minimal errors;
- Technological diversity: The review showcases different AR tools and methods, highlighting the diversity in technological approaches.

Sparwasser et al. [49] discuss AR and VR integration in surgery, highlighting their potential to improve clinical outcomes and surgical training. The study calls for rigorous clinical trials to evaluate these technologies.

The key insights are as follows:

- Enhanced training: AR and VR offer immersive training tools, improving educational methods for surgeons;
- Clinical validation: Rigorous trials are needed to validate the benefits of AR and VR in surgical practice;
- Future developments: Potential advancements include AR-based imaging overlays and expanded remote guidance through telementoring.

Ong et al. [50] examine the use of extended reality (ER) in ophthalmology, focusing on education, diagnostics, and therapeutics. Among the 87 studies reviewed, ER technologies show significant potential, despite the generally poor quality of the studies.

The key insights are as follows:

- Educational tools: ER technologies improve training in ophthalmology, offering better visualization and a better understanding of procedures;
- Diagnostic advances: Enhanced imaging capabilities support more accurate diagnostics and assessments of ocular diseases;
- Therapeutic applications: ER technologies could improve therapeutic outcomes, though higher quality comparative studies are needed.

Umana et al. [51] focus on subaxial cervical spondylodiscitis, particularly cases requiring three or more levels of cervical corpectomies. The review highlights AR’s role in complex spinal surgeries, showing mostly successful outcomes, despite the challenges.

The key insights are as follows:

- Complex surgeries: AR enhances precision in complex spinal surgeries, offering improved outcomes;
- Case studies: Practical insights from case studies demonstrate AR’s potential in real-world applications;
- Technological challenges: Addressing issues like spinal deformities and instability are crucial for effective AR use in spinal surgeries.

Doughty et al. [52] investigate the use of optical see-through head-mounted displays (OST-HMDs) for AR in surgery, analyzing 57 articles. The review focuses on orthopedic and maxillofacial surgery, highlighting challenges like system accuracy and technical difficulties.

The key insights are as follows:

- Head-mounted displays: OST-HMDs offer potential benefits in surgical navigation, improving precision and the user experience;
- System accuracy: Despite promising results, issues with accuracy and user perceptions need to be addressed;
- Technological advancements: Improvements in regard to interaction and perception are essential for the wider adoption of OST-HMDs in surgery.

Rodriguez Peñaranda et al. [53] investigate the role of artificial intelligence (AI) in renal cancer surgery training, focusing on advanced imaging to improve training and planning. The review analyzes 14 eligible studies, highlighting AI’s potential and the challenges.

The key insights are as follows:

- AI integration: AI enhances surgical training by improving preoperative preparation and intraoperative guidance;
- Ethical concerns: Addressing privacy, bias, and ethical issues are crucial for widespread AI adoption;
- Technological challenges: Improving real-time tracking and its integration with AR technologies is essential for optimizing AI’s role in surgery.

Colombo et al. [54] evaluate the 3D segmentation and visualization techniques for brain arteriovenous malformations (AVMs), highlighting the potential of machine learning for automatic segmentation and the need for better visualization tools.

The key insights are as follows:

- Segmentation techniques: Machine learning advancements improve automatic segmentation of brain AVMs;
- Visualization tools: Innovative methods are required to enhance the visualization and management of AVMs;
- Technological potential: Continued development could significantly improve the characterization and treatment of AVMs.

Checcucci et al. [55], in evaluating 3D printing and virtual imaging in kidney cancer surgery, find significant advantages in preoperative planning. Analyzing 29 studies, the review highlights the benefits and limitations of these technologies.

The key insights are as follows:

- Three-dimensional Printing: 3D printing provides benefits in simulations, patient counseling, and precise preoperative planning;
- Material limitations: Issues like cost and the quality of materials need to be addressed for broader application;
- Virtual imaging: Virtual imaging enhances the accuracy of preoperative planning and supports better surgical outcomes.

Unberath et al. [56] discuss image-based navigation in minimally invasive surgery, emphasizing the challenges in 2D/3D registration. The study highlights the potential of machine learning to address these challenges.

The key insights are as follows:

- Navigation techniques: AR and VR improve navigation and visualization during minimally invasive surgeries, enhancing precision and safety;
- Machine learning: Machine learning offers promising solutions for overcoming registration challenges and improving surgical outcomes;
- Future research: Continued research and development are needed to address the current limitations and fully realize the potential of these technologies in surgery.

#### 3.2.2. Comparative Analysis of the Systematic Reviews and Emerging Themes

Augmented reality (AR) is rapidly emerging as a transformative technology in the field of imaging, offering innovative solutions to enhance various aspects of these applications in the *health domain*. A series of systematic reviews shed light on distinct facets of AR applications in imaging, each focusing on a specific theme within the health domain. Table 3 reports on the specific themes addressed by each one of the systematic reviews.

However, it is possible to conduct a comparative analysis by exploring the intersections between these themes, thereby identifying commonalities, differences, and potential areas for synergy.

*Preoperative Planning and Simulation*

Sun et al. [41] focus on the revolutionary potential of AR in improving preoperative simulation, intraoperative navigation, and postoperative rehabilitation in hip surgery. Their systematic review emphasizes how VR and AR technologies can reduce complications, improve surgical success, and contribute to safer, more precise surgeries in hip procedures.

Dubron et al. [44], on the other hand, center their review on the preoperative planning of orbital fractures, highlighting the role of AR in improving surgical accuracy and precision. Their work underscores how AR facilitates better identification of anatomical structures and supports precise orientation and fixation of implants, albeit with a technical accuracy margin to consider.

*Intraoperative Guidance and Surgical Precision*

While Guha et al. [43] discuss the challenges in neurosurgery, they suggest that ongoing improvements in imaging, registration accuracy, display technology, and robotic integration could significantly increase the utility of AR. Their review explores the historical development and current applications of AR in neurosurgical procedures, highlighting its potential despite challenges, such as registration errors and depth perception issues.

Chidambaram et al. [47] also focus on neurosurgery, emphasizing the potential of AR to outperform traditional neuronavigation systems in improving surgical precision and guidance. Their systematic review underscores the promise of AR technology, but acknowledges challenges, such as related to accurate MRI brain segmentation and coregistration errors, that need to be addressed.

*Training and Education*

Kanschik et al. [42] and Sparwasser et al. [49] underline the value of AR in training medical staff, improving patient rehabilitation, and enhancing surgical training across various specialties. Kanschik et al.’s review emphasizes the usefulness of VR and AR for training ICU staff and assisting patients in intensive care units, while Sparwasser et al.‘s review discusses the growing body of research where AR and VR applications are finding their way into routine surgical practice.

Bosc et al. [48] categorize AR surgical applications into different types, emphasizing the accuracy of AR with minimal errors and its potential for hands-free and heads-up guided surgery. Their review provides insights into the various ways AR can be utilized in surgical procedures, ranging from heads-up guided surgery to digitally projecting images directly onto the patient.

*Integration with Advanced Imaging and Robotics*

Seetohul et al. [45] and Rodriguez Peñaranda et al. [53] discuss the integration of AR with surgical robotic and autonomous systems, and the potential of AI to refine surgical training and improve intraoperative guidance. Seetohul et al. evaluate surgical robots according to their hardware and computer vision capabilities, emphasizing the importance of advanced imaging techniques and deep learning algorithms in overcoming obstacles in surgery, while Rodriguez Peñaranda et al. investigate the role of AI in renal cancer surgery training, focusing on its application in advanced imaging to improve training and planning.

Rodler et al. [46] demonstrate the potential of combining various imaging modalities with robotic-assisted surgery, although prospective studies confirming the benefits are still in progress. Their review categorizes studies based on imaging of the primary tumor, the intraoperative detection of lymph nodes, and surgeon training, highlighting the potential of combining imaging modalities such as MRI and PET CT with robotic-assisted procedures.

*Challenges and Future Directions*

Various systematic reviews, as for example the ones reported in [50,51,52,54,55], highlight technical challenges such as system accuracy, user perception issues, and optimization difficulties with AR systems. These challenges underscore the need for further research and technological advancements to maximize the benefits of AR in surgery.

Sparwasser et al. [49] and Unberath et al. [56] call for rigorous clinical trials to evaluate AR technologies and suggest further research to address the current problems and explore the potential of machine learning in improving 2D/3D registration techniques. Their reviews emphasize the importance of addressing the challenges and advancing technologies to realize the full potential of AR in improving patient outcomes and surgical practices.

## 4. Discussion: *Illuminating the Highlights, Potential, Limitations, and Complementation of the Overview*

The umbrella review conducted on the systematic reviews focused on AR in digital imaging has allowed us to uncover the dominant themes within this field. Furthermore, it has highlighted the integration of AR with other technologies in this domain, showcasing its potential and the associated challenges. This comprehensive analysis not only identifies the key areas where AR is making significant strides, but also underscores the opportunities for future research and the hurdles that need to be addressed to fully leverage AR’s capabilities in digital imaging.

The section is divided into several paragraphs. Section 4.1 offers preliminary insights, presenting initial observations and understandings. In Section 4.2, various areas of potential that have emerged are explored, investigating potential avenues for further research or development. Section 4.3 identifies and discusses specific areas requiring deeper examination. Section 4.4 delves into discourse on the current status of regulatory integration and medical devices within the healthcare sector, providing a more comprehensive overview of the advancements in integration. Subsequently, the key message (Section 4.5) and limitations (Section 4.6) of the study are outlined.

### 4.1. Early Insights and Highlights

The integration of AR into the field of medical imaging boasts a rich history, dating back to 1995. However, it is within the past five years that we have observed a remarkable surge in interest and research output in this domain. This surge coincides with the onset of the COVID-19 pandemic, a period marked by unprecedented challenges and opportunities for technological innovation.

During the pandemic, there was a notable acceleration in the development and adoption of cutting-edge technologies across various sectors, particularly within healthcare. The urgency to find solutions for diagnosis, treatment, and remote patient care fueled significant investments and efforts in exploring the potential of AR [57], virtual reality (VR) [58], and artificial intelligence (AI) [59] to revolutionize healthcare delivery.

The pandemic served as a catalyst for advancements not only in diagnostic imaging, but also in a broader spectrum of healthcare applications. AR emerged as a promising tool for enhancing medical visualization, surgical planning, and training, among other applications.

Furthermore, the convergence of AR with AI and VR technologies opened up new vistas for personalized medicine, remote patient monitoring/telerehabilitation integrated with robotics, and immersive healthcare experiences [60,61]. These synergistic technologies empowered healthcare professionals to remotely assess and treat patients, design personalized treatment plans, and even conduct virtual surgeries, thereby overcoming geographical barriers and enhancing access to quality care.

In examining publication trends, it becomes evident that the surge in AR-related research during the pandemic extended beyond imaging alone. Rather, it permeated various facets of healthcare, reflecting a broader trend toward the integration of advanced technologies to address evolving healthcare needs.

**Figure 5:**
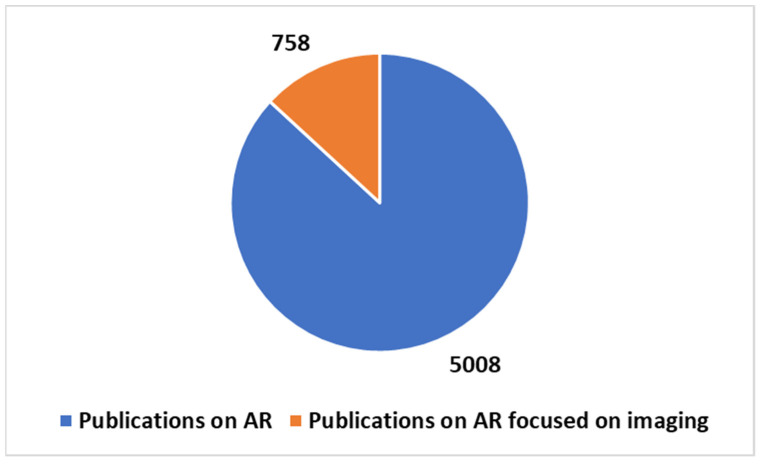
Trends of the publications on AR compared to the publications on AR focused on imaging.

**Figure 6:**
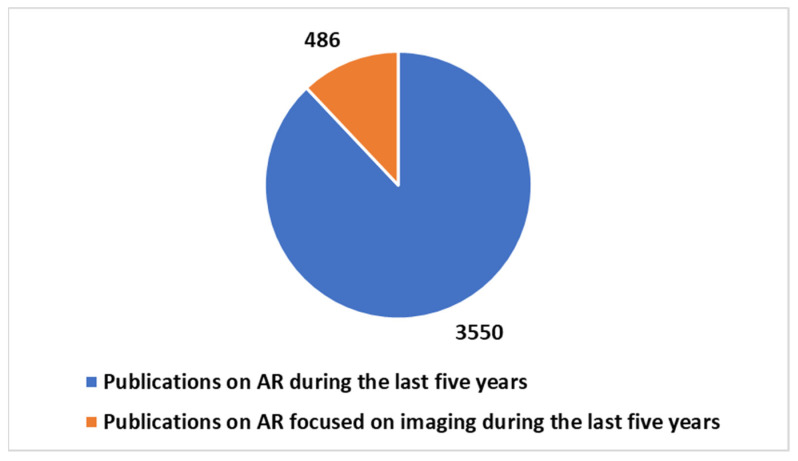
Trends of the publications on AR over the last five years compared to the publications on AR focused on imaging in the same period.

**Figure 7:**
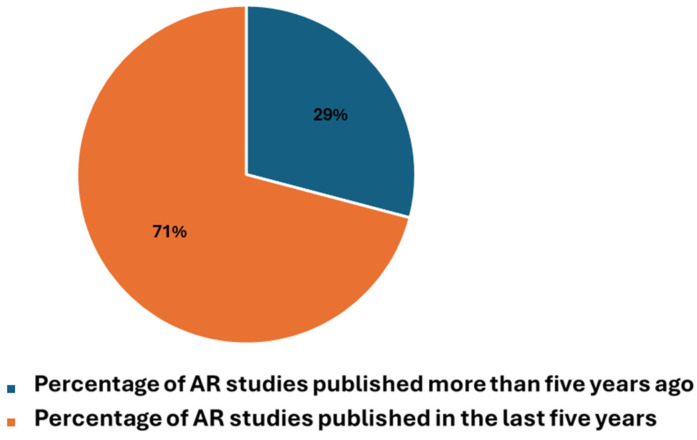
Trends of the publications on AR during and before the last five years.

According to a search on PubMed, using the key in Box 1 position 2 (Figure 5, Figure 6 and Figure 7), out of a total of 5008 publications on AR, approximately 15% (758 publications) focused significantly on imaging. Focusing on the last five years, AR-related publications constituted 70.9% of the total, with 486 publications dedicated to imaging, representing around 14% of the total publications during that period, a value that remained relatively stable. These percentages underscore the substantial growth and impact of AR in healthcare, particularly during the COVID-19 pandemic.

The evolving themes, shifting research trends, and burgeoning wealth of knowledge within the field provide the impetus and rationale for our overview study. As outlined in the literature [62,63], an overview entails a comprehensive synthesis of findings from multiple systematic reviews on a specific subject. Our decision to undertake an overview is firmly grounded in the domain of systematic reviews, recognized for their meticulous analyses and scientific rigor.

By immersing ourselves in these studies, our aim is to offer a holistic overview of the existing research landscape, with a particular emphasis on systematic reviews. Systematic reviews systematically collect and evaluate evidence, contributing to a nuanced understanding of the subject matter. This comprehensive approach not only illuminates areas ripe for exploration, but also identifies specific avenues warranting further investigation, underscoring global areas requiring heightened research focus and attention.

Moreover, our study, in contrast to other reviews/systematic reviews in this field that have focused on specific and narrow themes, adds value by delving into themes and trends from a broader perspective. This approach unveils overarching patterns and themes with significant implications for further scholarly inquiry, providing important insights for scholars.

### 4.2. Emerging Potential

#### 4.2.1. General Potential in Imaging Integration

AR technology stands at the forefront of surgical innovation, poised to potentially revolutionize the field and elevate patient care standards [41,42,43,44,45,46,47,48,49,50,51,52,53,54,55,56]. By seamlessly integrating digital information into surgical environments, AR equips surgeons with unparalleled levels of visualization, spatial awareness, and precision.

Among its myriad potential benefits, AR’s integration with imaging technologies enhances visualization and spatial awareness during surgical procedures [41,43,45,47,50,52,53,54]. By overlaying critical anatomical information onto the surgical field in real-time, AR has the potential to empower surgeons to navigate complex structures confidently, reducing the risk of inadvertent damage and facilitating more precise interventions.

Moreover, AR shows promising potential to facilitate comprehensive preoperative planning and simulation by integrating patient-specific imaging data into a 3D virtual environment [41,44,46,49,51,55,56]. This could allow surgeons to meticulously plan procedures, simulate scenarios, and develop optimal strategies before entering the operating room, thereby enhancing preparedness and improving patient outcomes.

During surgery, AR-based navigation systems have the potential to provide real-time guidance, enhancing precision and efficiency [41,43,45,47,50,52,54,56]. By overlaying virtual markers onto the surgical field, AR assists in accurately locating target structures, planning incisions, and optimizing instrument positioning, ultimately streamlining procedures and reducing the risk of complications. Furthermore, AR supports surgical education and training, offering immersive learning experiences for trainees [41,44,46,49,51,55]. Virtual reality simulators and augmented educational platforms allow trainees to practice procedures in a risk-free environment, receive immediate feedback, and refine their skills, ultimately enhancing competency and patient safety.

Beyond clinical practice, AR enhances patient engagement and communication by providing interactive visualizations of medical conditions and treatment plans [41,43,45,47,50,52,54,56]. Surgeons can utilize AR to educate patients, demonstrate interventions, and address concerns, fostering shared decision-making, satisfaction, and treatment adherence. Moreover, AR enables personalized medicine and surgical precision by integrating patient-specific data into workflows [41,44,46,49,51,55]. Surgeons can tailor treatment strategies based on individual anatomy and preferences, optimizing outcomes and minimizing risks, thus ensuring accurate and effective interventions tailored to each patient’s needs.

Table 3 reports the emerging potential in the referenced studies.

#### 4.2.2. Enhancing Integration Potential with Other Innovative Technologies in Imaging Applications

The integration of AR technology with other cutting-edge innovations such as AI, robotics, and VR in virtual and augmented reality (VAR) environments presents a compelling frontier, as suggested by the analyzed studies [41,42,43,44,45,46,47,48,49,50,51,52,53,54,55,56]. While these integrations are briefly mentioned across the studies, a closer examination reveals specific instances where this fusion is explored in greater depth.

VAR technologies, for instance, offer promising avenues for enhancing the precision and safety of hip-related surgeries when integrated with robotic systems [41]. By amalgamating VAR with robotics, surgeons gain access to real-time imaging and navigation data, facilitating optimized surgical workflows, improved instrument positioning, and precise implant placement. The complementarity of robotic-assisted platforms with VAR enhances dexterity and control during procedures, ultimately leading to superior surgical outcomes and reduced complication rates.

In the domain of intensive care medicine, the fusion of VAR with AI holds transformative potential for training, procedural planning, and patient care [42]. AI algorithms analyze patient data, aiding in decision-making and outcome prediction, while VAR technologies create immersive training environments and offer real-time guidance during complex procedures like cardiopulmonary resuscitation and vascular punctures.

Neurosurgery stands to significantly benefit from the integration of VAR with robotics and AI algorithms [43]. By incorporating robotic platforms with VAR-based navigation tools, surgeons can achieve unprecedented levels of precision and accuracy in various procedures, from tumor resections to intracranial interventions and spinal surgeries. AI algorithms further enhance surgical planning and decision-making, while VAR technologies provide invaluable real-time visualization and guidance, ultimately enhancing surgical outcomes and patient safety.

Similarly, in the management of orbital fractures, the convergence of VAR with AI and robotics streamlines preoperative planning and intraoperative guidance [44]. AI algorithms analyze patient imaging data and simulate surgical procedures, while VAR technologies empower surgeons with a comprehensive visualization of complex anatomical structures and optimal surgical approaches. Robotic systems, in turn, offer precise instrument control and navigation assistance, leading to more accurate fracture reduction and improved aesthetic results.

Precision surgery and training in renal cell carcinoma (RCC) can also harness the potential of VAR integration with robotics and AI [53]. AI algorithms analyze imaging data and tumor characteristics to tailor surgical approaches, while VAR technologies provide immersive training environments and real-time visualization of tumor anatomy. Robotic systems complement these advancements by offering dexterity and precision for tumor resection and organ preservation, ultimately optimizing surgical outcomes in RCC cases. Table 4 details this integration with other innovative technologies in imaging applications.

### 4.3. The Need for Broader Investigations

#### 4.3.1. The Need for Broader Investigations in Specific Areas

The strategic integration of augmented reality (AR) and related technologies within various surgical specialties encapsulates a dynamic and multifaceted discourse, delineating the potential benefits and inherent challenges of adopting these cutting-edge tools. Across fields such as orthopedics, neurosurgery, ophthalmology, and beyond, researchers and clinicians are at the forefront of exploring how AR can redefine surgical paradigms, from enhancing visualization to refining surgical precision, streamlining education and training and, ultimately, optimizing patient outcomes [41,43,45,47,49,53,54,55,56].

A pivotal area of inquiry lies in preoperative planning and simulation, where AR facilitates the immersive exploration of patient anatomy and enables surgeons to rehearse procedures within a virtual environment prior to stepping into the operating room [41,44,49,54,55,56]. By seamlessly overlaying patient-specific imaging data onto the surgical field, AR platforms empower surgeons to craft meticulous surgical strategies, thereby mitigating procedural risks and augmenting patient outcomes [41,44,49,54,55,56]. However, the validation of AR-assisted preoperative planning, especially in intricate procedures like orbital fractures and spinal surgeries, necessitates further research to ascertain its accuracy and clinical efficacy [44,51].

Intraoperative navigation and guidance represent another pivotal domain [41,43,45,47,49,53], where AR-based systems furnish real-time assistance to surgeons, heightening surgical precision and efficiency. Through the projection of virtual markers and trajectories onto the surgical field, AR aids in the precise localization of target structures and the optimization of instrument placement. While studies have showcased promising outcomes in enhancing surgical efficacy and reducing operative duration [41,43,45,47,49,53], addressing challenges such as those related to accurate brain segmentation and hardware limitations remains imperative for scaling AR utilization across specialties [43,47,52].

Education and training undergo a transformative shift with the integration of AR technology [41,42,44,49,50,55], as virtual reality simulators and augmented educational platforms offer immersive learning experiences for trainees. AR-driven training modules foster skill development and procedural proficiency among trainees, thus fortifying patient safety and outcomes [41,42,44,49,50,55]. Nonetheless, the efficacy of AR-driven educational tools and their seamless integration into existing training frameworks necessitate rigorous evaluation and validation through clinical studies [42,50].

Furthermore, personalized medicine and surgical precision emerge as pivotal realms where AR demonstrates significant promise [41,44,49,54,55,56]. By amalgamating patient-specific data and anatomical models into the surgical workflow, AR facilitates tailored treatment strategies that optimize outcomes and mitigate risks [21,24,29,34,35,36]. Real-time feedback on instrument positioning and tissue manipulation further enhances surgical precision, paving the way for more accurate interventions. Nonetheless, addressing technical accuracy concerns and validating the clinical benefits of AR systems through ongoing research and development endeavors remain paramount [41,44,49,54,55,56]. In essence, the discourse surrounding AR in surgery oscillates between excitement about its transformative potential and the imperative for rigorous evaluation and validation [41,45,53,56]. Collaborative endeavors among clinicians, engineers, and researchers are indispensable in surmounting technical challenges, validating clinical benefits, and ensuring the seamless integration of AR into surgical practice. As AR technology evolves, it harbors the potential to revolutionize surgical practice across specialties, catalyzing improved patient outcomes and shaping the future landscape of surgery.

Table 5 reports the specific emerging suggestions for broader investigation.

#### 4.3.2. Key Focus Areas for Broader Exploration in AR Applications in Imaging

In addition to the specific areas of focus outlined in the referenced studies, it is crucial to identify common/transversal themes that necessitate broader investigation. These common areas span across multiple disciplines and underscore the need for deeper exploration, as follows:

*The integration of VR, AR, and robotics:* Various studies underscore the potential synergy between virtual reality (VR), augmented reality (AR), and robotics in diverse surgical procedures, with a particular emphasis in studies focused on hip-related surgeries [41], intensive care medicine [42], neurosurgery [43], orbital fracture management [44], and kidney cancer surgery [53]. Delving into the combined effects and challenges of integrating these technologies could unlock pathways to more efficient and effective surgical interventions.

*Clinical outcomes and comparative studies:* While the benefits and application of AR are extensively discussed across different medical fields [41,42,43,44,45,46,47,48,49,50,51,52,53,54,55,56], there remains a consistent need for comparative studies evaluating their impact on clinical outcomes. Such studies, examining parameters like complication rates, surgical success rates, patient recovery, and cost effectiveness, offer valuable insights into the superiority of these technologies over traditional methods, guiding decision-making in clinical practice.

*The optimization of surgical workflows:* The integration of VR, AR, and robotics aims to optimize surgical workflows by offering real-time guidance, precise navigation, and enhanced visualization [41,42,43,44,45,46,47,48,49,50,51,52,53,54,55,56]. Investigating the impact of these technologies on streamlining surgical procedures, reducing operative time, and enhancing overall efficiency, is pivotal for advancing surgical practices and ensuring optimal patient outcomes.

*Technological challenges and solutions:* Despite the promising potential of VR, AR, and robotics in medicine, significant technological challenges persist [41,42,43,49,50,51,52,56], including registration errors, depth perception issues, hardware limitations, and data privacy concerns. Addressing these challenges necessitates further research into developing advanced algorithms, enhancing hardware capabilities, and addressing ethical considerations to maximize the benefits of these technologies in clinical settings.

By addressing these common/transversal areas through comprehensive research and collaborative efforts, we can advance our understanding of the potential benefits and challenges associated with the integration of VR, AR, and robotics in surgical practice, ultimately enhancing patient care and surgical outcomes. Table 6 reports the emerging common/transversal areas where the need for deeper exploration arises.

The analysis underscores a notable gap in systematic reviews, particularly in sectors of imaging like digital dermatology and medical tattooing [64,65]. These areas have yet to attain sufficient robustness for inclusion in broader thematic discussions, indicating a need for more exhaustive examination within existing research.

AR presents a promising avenue for enhancing medical and professional practices within these sectors. In digital dermatology, AR could aid physicians in diagnosing and treating skin conditions with greater accuracy and efficiency. By overlaying virtual information onto skin images, AR provides valuable insights for diagnosis and monitoring.

Similarly, in medical tattooing, AR could serve as a visual guide during operative procedures, offering crucial support to professionals in ensuring precision, particularly in surgical or complex interventions.

The application of AR technology has the potential to enhance the efficiency, accuracy, and safety of medical practices in imaging sectors that have been addressed less by systematic reviews. This underscores the importance of further exploration and development of AR in specific areas like digital dermatology and medical tattooing.

Another gap warranting targeted discussion is regulatory integration, which is subject to diverse national and supranational regulations, particularly in the realm of medical devices. Addressing regulatory discrepancies and challenges across jurisdictions is essential for seamless integration within the healthcare domain.

This involves navigating compliance with national laws and international standards, ensuring the interoperability and safety of medical devices, and fostering a regulatory environment that encourages innovation, while prioritizing patient well-being. Thus, comprehensive dialogue on regulatory harmonization becomes imperative to facilitate the smooth functioning of healthcare systems and promote optimal patient outcomes.

### 4.4. Comprehensive Discussion on Harmonization and Alignment of Regulation

The convergence of AR with the healthcare sector, particularly in the realm of medical imaging, hinges significantly on the standardization of operational regulations and the harmonization of practices within the medical devices industry. Adhering to these regulatory frameworks is crucial for ensuring the seamless integration of AR technologies into healthcare workflows within the broader health domain.

#### 4.4.1. Standardization of Imaging in Digital Pathology and Radiology and the Impact on AR Integration

Within the field of imaging, there are two notable focal points or polarities. The *first polarity* revolves around digital radiology, which encompasses the use of digital technology for capturing, storing, and interpreting medical images, such as X-rays, CT scans, and MRI scans. This digitization of radiological processes has revolutionized diagnostic imaging, offering greater precision, efficiency, and accessibility for healthcare professionals.

On the other hand, *the second polarity* centers on digital pathology, which involves the digitization of tissue samples and microscopic images for diagnostic analysis. Digital pathology enables pathologists to leverage advanced image analysis algorithms, telepathology solutions, and collaborative platforms to enhance accuracy, speed, and collaboration in diagnosing diseases and guiding treatment decisions.

These *two polarities* within the imaging domain represent distinct but complementary aspects of modern healthcare delivery. By embracing AR technology and integrating it with digital radiology and digital pathology workflows, healthcare providers can unlock new possibilities for enhanced visualization, diagnostic accuracy, surgical planning, medical education, and patient engagement. However, achieving this integration necessitates careful attention to regulatory standards, interoperability requirements, data security considerations, and user training, to ensure the safe and effective adoption of AR solutions in clinical practice.

Radiology has indeed undergone a remarkable evolution, driven by the digitization processes catalyzed by the Digital Imaging and Communications in Medicine (DICOM) standard [66]. This evolution has seamlessly integrated radiology into the broader landscape of digital health, marking a fundamental shift from analog to digital methodologies. However, it is noteworthy that this integration has progressed at a different pace compared to fields like digital pathology, where DICOM adoption has faced delays.

While radiology swiftly embraced DICOM standardization, leading to expanded data exchange capabilities and the proliferation of remote diagnostics, digital pathology faced delays in this integration with the DICOM Whole Slide Image [67,68,69] and this had a consequent effect on its integration with the innovative technologies in the health domain.

It must also be considered that the two fields differ greatly in their approach. Digital radiology is oriented toward the imaging of organs and/or functionality. Digital pathology deals with the imaging of tissues and cells. DICOM has played an important role in integrating digital radiology into digital healthcare, hospital processes, and networks [70,71]. The increasingly advanced capabilities of technology have also pushed voxels toward miniaturization, which indirectly, thanks to DICOM, has significantly boosted integration with AR/VR [72].

In summary, while digital radiology has swiftly integrated into the digital healthcare landscape, digital pathology has faced challenges in regard to standardization and integration. Recognizing these differences is essential for fostering comprehensive and effective digital transformation in healthcare, ensuring that all imaging modalities contribute synergistically to improved patient care and outcomes.

#### 4.4.2. Trends in the Integration of Augmented Reality Medical Devices within the Health Domain

##### *Trends* 

The different worlds and evolution of AR and VR are deeply interconnected in the healthcare industry.

The dominance of the augmented reality (AR) segment in the healthcare industry’s AR and VR sector is significant, capturing a major share of approximately 60.20% [22]. This dominance is attributed to the widespread use of head-mounted devices and the increasing adoption of smart glasses, which facilitate the presentation of 3D images and provide enhanced views of the environment. The rising demand for augmented reality stems from its potential to improve treatment for various diseases, a trend that has seen considerable growth in recent years. Technological advancements play a pivotal role in driving industry growth, while investments from public firms and industry players further contribute to its expansion.

Additionally, the VR segment is poised for rapid growth in the foreseeable future. Virtual reality technology enables users to interact with immersive three-dimensional environments, offering promising potential for rehabilitation and pain management. Virtual exposure therapy, utilized in treating phobias such as a fear of heights, driving, claustrophobia, and post-traumatic stress disorder, underscores the versatility and effectiveness of VR applications. The increasing prevalence of neurological disorders and the growing demand for innovative diagnostic methods are key drivers propelling the growth of the VR segment within the healthcare industry.

This union between AR and VR must be taken into account, particularly when discussing integration in imaging. From the systematic review studies analyzed, from the studies examined in the introductory hypotheses, and in the complementary discussions, there is an emphasis on the predominant contribution of diagnostic and functional organ imaging supported, as reported above, by digital radiology, with rare exceptions, such as in AR applied in virtual Slab [23] or in teledermatology [24].

##### *Approval as a Medical Device* 

The integration of AR and VR within the healthcare domain, especially in imaging driven by digital radiology, reflects a growing trend toward genuine integration. This trend is evident in the approvals of medical devices within this domain. Certification processes are essential for integrating devices into the *healthcare domain*, ensuring compliance with regulatory standards before market introduction and utilization.

**Figure 8:**
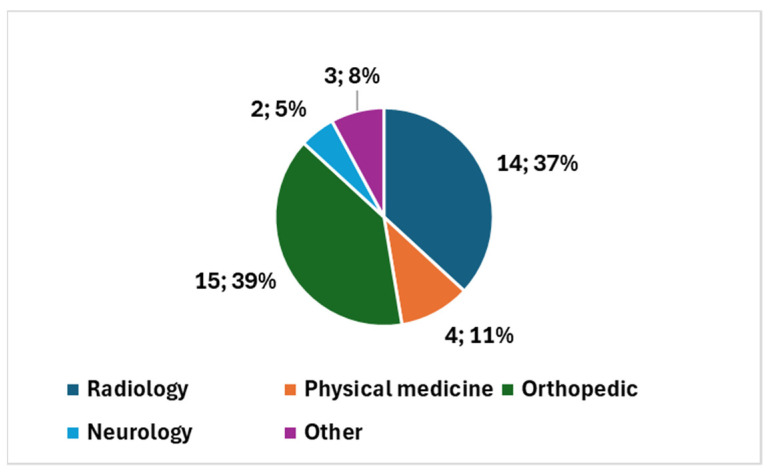
FDA-approved AR/VR devices divided by category.

AR devices intended for routine medical use in the health domain must undergo a certification process as medical devices [73]. As seen in [72,73], the FDA has developed a dedicated interface for AR/VR medical devices and structured communication with professionals and citizens [74,75]. Among the FDA-approved medical devices, radiology holds a significant position and is visible online (Figure 8), representing a notable percentage. Further analysis of Figure 8 also indicates that the radiology component plays a translational role by contributing to imaging in other areas such as orthopedics.

Based on additional insights [72,73], it is evident that such medical devices are classified with a low risk class (Class I), limiting their use to non-diagnostic applications [73,76,77,78], although they are useful, for example, in providing complementary informational content and, in some cases, enabling patients, whose attention to these issues is growing [79], to provide more informed consent.

In Europe, these devices are considered to be Software as a Medical Device, based on the regulations in this field [80,81]. According to a recent study [82], Europe is reportedly lagging behind in this area for several reasons. Among these reasons, the authors identify both the transition processes from the previous regulations [83] and the handling of these software technologies in the medical field [84,85], which significantly differ from those made for use by consumers [86,87].

The approval by Health Canada [88] of one of these AR/VR devices in risk class 2 has caused a stir, due to its prospective implications.

#### 4.4.3. Key Considerations on Harmonization and Alignments in Terms of Regulation

The discussion on harmonization and the alignment of regulations highlights the crucial role of standardization in integrating AR technology within the healthcare sector, especially in medical imaging. This integration requires adherence to regulatory frameworks to ensure the seamless incorporation of AR technologies into healthcare workflows [73].

Digital radiology and digital pathology represent two distinct but complementary aspects of modern healthcare delivery, both essential for accurate diagnosis and treatment. While digital radiology has swiftly integrated into the digital healthcare landscape, digital pathology has faced challenges in standardization and integration [66,67,68,69]. While digital radiology swiftly embraced DICOM standardization, digital pathology encountered challenges due to specialized adaptations and longer release times [67,68,69]. This difference in the pace of integration underscores the diverse nature of imaging modalities within healthcare and highlights the need for tailored approaches to integration and standardization. Despite these challenges, both digital radiology and digital pathology play crucial roles in modern healthcare delivery, emphasizing the importance of comprehensive and effective digital transformation in healthcare [73,88]. However, there is currently a notable absence of medical devices specialized in digital pathology, among those equipped with AR technology (Figure 8).

The integration of AR and VR within the healthcare domain, particularly in imaging driven by digital radiology, reflects a growing trend toward genuine integration [72,73]. Certification processes are crucial for integrating devices into the healthcare domain, ensuring compliance with regulatory standards before market introduction [73]. However, except for Health Canada [88], which recently classified one of these devices as class 2, currently these devices have applications associated with low-risk classes and are not directly involved in diagnostic processes [77,78].

It is therefore crucial to approach the overview with objectivity, ensuring a balanced and thorough assessment of the potential and challenges presented by AR/VR integration within the healthcare domain [76,77,78]. Objective analysis allows for a comprehensive understanding of the potential benefits and risks associated with these technologies, guiding informed decision-making and facilitating their safe and effective implementation in clinical practice.

### 4.5. Takeaway Message

Significant initiatives have focused on integrating AR with imaging technologies, with a notable acceleration during the COVID-19 pandemic. This period highlighted significant development potential alongside specific areas in need of improvement and further scientific exploration, as well as aspects not thoroughly investigated enough to establish consolidated themes. Moreover, there is a widespread demand for thorough investigation into the synergies between integrating VR, AR, and robotics, clinical outcomes through comparative studies, the optimization of surgical workflows, and additional solutions to address technological challenges. It is essential to emphasize that further insights and advancements in the realm of medical devices and regulatory frameworks are necessary.

### 4.6. Limitations

The study is based on an umbrella review, which is an overview of systematic reviews. The analysis of systematic reviews is strategic, aimed at uncovering emerging themes in a bottom-up manner. Expanding the study to address these emerging themes individually can allow for a detailed comparison of the AR methodologies used, critically comparing them within each theme of interest. Focusing on national and international databases can provide a more detailed understanding of the local state of integration based on internal regulations and applied legislation.

## 5. Conclusions

This study unveils a comprehensive exploration into various facets of AR integration within imaging technologies. Early insights illuminate a significant surge in AR-related research, particularly amidst the challenges and potential presented by the COVID-19 pandemic. This surge underscores AR’s transformative potential, from enhancing visualization and spatial awareness in surgical procedures to providing immersive educational experiences for trainees.

Furthermore, our overview delves into the emerging potential, accentuating AR’s role in improving surgical precision, patient engagement, and personalized medicine. The integration of AR with other innovative technologies like VR, AI, and robotics opens new avenues for enhancing patient care and surgical outcomes. We have identified specific areas warranting deeper examination, such as preoperative planning and simulation, intraoperative navigation, and education and training, underscoring the imperative for rigorous evaluation and validation.

Moreover, there is a widespread call for in-depth examinations regarding the synergies between integrating AR, VR, and robotics, clinical outcomes through comparative studies, and optimizing the surgical workflow, alongside addressing the technological challenges. Additionally, our analysis highlights gaps in existing systematic reviews, particularly in sectors like digital dermatology and medical tattooing, indicating untapped potential for AR applications. The systematic reviews also overlook crucial regulatory and developmental aspects, particularly concerning medical devices, in their examination of the integration of AR technology into healthcare. These aspects encompass compliance with standards, safety regulations, risk management, clinical validation, and developmental processes like design and engineering. By incorporating these considerations, researchers can provide a more comprehensive understanding of the challenges and potential associated with integrating AR into clinical practice, informing stakeholders about the regulatory requirements and developmental considerations necessary for successful implementation.

By bridging these gaps, AR has the potential to significantly enhance the efficiency, accuracy, and safety of medical practices in these sectors.

In conclusion, our overview provides insights into the evolving landscape of AR integration with imaging technologies, emphasizing the ongoing need for continued research, development, and focus on the regulation issues (also embedding medical device legislative frameworks) to fully realize its transformative potential.

## Figures and Tables

**Table 1:** Studies with a focus on the two areas of interest: (a) diagnostic imaging and (b) AR integration.

Review Study	Focus on Diagnostic Imaging	Focus on AR Integration
*Sun et al. (2023)* [41]	The study delves into the transformative role of virtual reality (VR) and augmented reality (AR) imaging technologies in hip-related surgery. It reviews 40 studies, showcasing their applications in preoperative simulation, intraoperative navigation, and postoperative rehabilitation. While highlighting their potential to enhance surgical precision and safety, it emphasizes the necessity for more comparative research to accurately gauge their clinical efficacy and cost effectiveness.	The study centers on augmented reality (AR) integration in hip-related surgery. Examining 40 studies, it assesses AR’s utility in preoperative simulation, intraoperative navigation, and postoperative rehabilitation. Despite showing promise for enhancing surgical precision and safety, further comparative research is crucial to fully understand AR’s clinical efficacy and cost effectiveness.
*Kanschik et al. (2023)* [42]	The study delves into the application of virtual reality (VR) and augmented reality (AR) in intensive care medicine, through a systematic review of 59 studies. It highlights how these technologies assist healthcare providers in training, planning procedures, and aiding patients’ well-being within the ICU setting. Despite variations in study design, VR and AR show promise in improving care practices, with the potential for further development and increased utilization in healthcare.	The study examines the integration of augmented reality (AR) in intensive care medicine, reviewing 59 studies to explore possible applications. AR assists healthcare providers in training, planning procedures, and supporting patients within the ICU setting. Despite variations in study design, AR demonstrates potential for enhancing care practices, indicating opportunities for further development and expanded integration in healthcare settings.
*Guha et al. (2017)* [43]	The systematic review investigates the evolution and current application of augmented reality (AR) in neurosurgery, a field known for its advancements in image-guided surgery. Despite challenges, such as registration errors and impaired depth perception, AR shows promise in accurately overlaying three-dimensional datasets in the surgical field. Future advancements in imaging, registration, display technology, and robotics are expected to further enhance AR’s role in improving surgical outcomes in neurosurgery.	The systematic review focuses on the application of augmented reality in neurosurgery, a field renowned for pioneering image-guided surgery. It assesses current neurosurgical AR applications, highlighting challenges like registration errors and impaired depth perception. Despite obstacles, AR’s ability to overlay three-dimensional datasets accurately signifies its potential in enhancing surgical precision. The review anticipates future advancements in imaging, registration, and display technology, underscoring AR’s promising role in neurosurgical operating rooms.
*Dubron et al. (2023)* [44]	The systematic review examines the utilization of extended reality, encompassing augmented reality (AR), mixed reality (MR), and virtual reality (VR), in preoperative planning for orbital fractures. It contrasts extended reality with conventional planning techniques, focusing on computer-aided surgical simulation, patient-specific implants (PSIs), and fracture reconstruction using computed tomography data. The review underscores AR’s role in enhancing accuracy and precision during orbital fracture management, particularly in guiding incisions, identifying anatomical tissues, and facilitating intraoperative imaging enhancement. Furthermore, it highlights VR-based educational tools for offering enhanced visualization and comprehension of craniofacial trauma compared to traditional imaging approaches.	The systematic review specifically examines the role of augmented reality (AR) in preoperative planning for orbital fractures, alongside mixed reality (MR) and virtual reality (VR). It compares AR with conventional planning techniques, emphasizing its contributions to computer-aided surgical simulation, patient-specific implants (PSIs), and fracture reconstruction based on computed tomography data. Notably, AR is highlighted for its ability to enhance accuracy and precision in orbital fracture management, guiding incisions, identifying anatomical tissues, and improving intraoperative imaging. Additionally, the review acknowledges AR’s potential in offering real-time visualization and precise positioning of implants during surgery, demonstrating its value in optimizing surgical outcomes.
*Seetohul et al. (2023)* [45]	The paper provides a systematic review of surgical robotic platforms, focusing on integrating augmented reality (AR) to enhance interventions. It addresses challenges like tool placement accuracy and depth perception in medical imaging. Analyzing robots such as Novarad and SpineAssist, it identifies shortcomings in optimization algorithms and proposes solutions for tool-to-organ collision detection. Despite challenges, the study suggests promising results in reducing occlusion and end-effector collisions, supporting AR’s potential in future surgical applications.	The paper systematically reviews the integration of augmented reality (AR) in surgical robotic platforms, aiming to enhance surgical interventions. It identifies challenges like tool placement accuracy and depth perception in medical imaging, proposing solutions to optimize AR technology. Analyzing robots such as Novarad and SpineAssist, it highlights AR’s role in improving the user’s perception of the augmented world. Despite challenges, the study indicates promising results, supporting AR’s potential to revolutionize surgical procedures in the future.
*Rodler et al. (2023)* [46]	The study provides a comprehensive analysis of new imaging technologies in robotic-assisted radical prostatectomy (RARP) for prostate cancer management. Through a systematic review of 46 studies, it highlights the use of imaging for primary tumor detection, intraoperative lymph node detection, and surgeon training. The feasibility of combining imaging technologies, such as MRI, PSMA-PET CT, and intraoperative dyes, has been demonstrated, but prospective confirmation of improved surgical outcomes is ongoing.	The study focuses on the integration of new imaging technologies, including augmented reality (AR), in robotic-assisted radical prostatectomy (RARP) for prostate cancer management. Through a systematic review of 46 studies, it examines the role of AR in primary tumor detection, intraoperative lymph node detection, and surgeon training. While the feasibility of combining AR with imaging modalities like MRI and PSMA-PET CT has been demonstrated, ongoing research aims to confirm their prospective impact on surgical outcomes.
*Chidambaram et al. (2021)* [47]	The systematic review delves into the role of augmented reality (AR) technology in neurosurgery, aiming to enhance intraoperative visualization and guidance beyond traditional neuronavigation. Through the analysis of 54 articles, it explores how AR has been integrated into clinical practice in both brain and spine subspecialties. Despite its potential benefits, challenges such as accurate brain segmentation, accounting for brain shift, and reducing coregistration errors need to be addressed for widespread adoption. Additionally, the study suggests future avenues for combining AR with multimodal imaging techniques and artificial intelligence to further improve its impact in neurosurgery.	The systematic review focuses on augmented reality (AR) technology in neurosurgery, aiming to enhance intraoperative visualization and guidance beyond traditional neuronavigation. Analyzing 54 articles, it explores AR’s integration into clinical practice in brain and spine subspecialties. While acknowledging potential benefits, the study highlights challenges like accurate brain segmentation and coregistration errors. It also proposes future research directions, suggesting the combination of AR with multimodal imaging techniques and artificial intelligence to enhance its impact in neurosurgery.
*Bosc et al. (2019)* [48]	The systematic review classifies augmented reality (AR) applications in maxillofacial surgery, distinguishing them from other virtual imaging procedures. Thirteen publications were analyzed, with five describing hands-free and heads-up AR approaches using smart glasses or headsets combined with tracking. Most reported minimal error (<1 mm) between virtual models and patients. AR during surgery is categorized into heads-up guided surgery with or without tracking, guided surgery using semi-transparent screens, digital projection onto patients, and digital data transfer to monitor displays.	The systematic review specifically focuses on augmented reality (AR) applications in maxillofacial surgery, aiming to differentiate them from other virtual imaging procedures. Thirteen publications were analyzed to classify AR methods, with five describing hands-free and heads-up AR approaches using smart glasses or headsets combined with tracking. The study categorizes AR during surgery into four types based on guidance techniques and visualization methods. Most publications reported minimal error between virtual models and patients, highlighting the potential of AR in enhancing surgical precision.
*Sparwasser et al. (2018)* [49]	The article discusses the current and future applications of virtual reality (VR) and augmented reality (AR) in surgery, emphasizing the potential clinical improvements. Through systematic literature research and analysis of investment trends, it highlights the increasing integration of AR and VR technologies into surgical practice. Key applications include intraoperative overlap with radiological imaging, telementoring, and surgical education. While promising, the benefits of AR and VR for clinical endpoints are yet to be fully understood and require rigorous examination through clinical trials. Physicians are expected to play a crucial role in leveraging these technologies for patient benefit in surgery.	The article assesses the current and future applications of augmented reality (AR) in surgery, alongside virtual reality (VR). Through systematic literature research and analysis, it highlights the increasing integration of AR into surgical practice. Key areas of focus include intraoperative overlap with radiological imaging, telementoring, and surgical education. However, the article also acknowledges the need for rigorous clinical trials to fully understand the benefits of AR for clinical endpoints. Physicians are expected to play a pivotal role in leveraging AR technology for patient benefit in surgery.
*Ong et al. (2021)* [50]	The systematic review explores the utility of extended reality (XR) in ophthalmology, covering education, diagnostics, and therapeutics. Out of 12,490 records, 87 met the eligibility criteria, with most studies focusing on education. While XR shows promise, the majority of studies were of poor quality, and only a few addressed validity evidence. Surgical simulators were found to improve performance and reduce complications, while ophthalmoscopy simulators enhanced clinical skills. In diagnostics, XR platforms demonstrated a proof-of-concept in presenting ocular imaging data and assessing patients with ophthalmic diseases. In therapeutics, heads-up surgical systems showed comparable outcomes compared to conventional surgery. However, more high-quality comparative studies are needed to fully evaluate XR’s role in ophthalmic practice.	The systematic review investigates the utility of extended reality (XR), which includes augmented reality (AR), in ophthalmology, particularly in education, diagnostics, and therapeutics. Out of 12,490 records, 87 studies met the eligibility criteria, with the majority focusing on education. While promising, most studies were of poor quality, and only a few addressed validity evidence. Surgical simulators were found to improve performance and reduce complications, while ophthalmoscopy simulators enhanced clinical skills. In diagnostics, XR platforms demonstrated a proof-of-concept in presenting ocular imaging data and assessing patients with ophthalmic diseases. However, more high-quality comparative studies are needed to fully assess the role of XR, including AR, in ophthalmic practice.
*Umana et al. (2023)* [51]	The study investigates the management of subaxial cervical spine spondylodiscitis, focusing on ≥three-level cervical corpectomies. A literature review and an emblematic case presentation are conducted. Thirteen papers were selected, with 28 patients treated with ≥three-level corpectomy. A combined anterior and posterior approach was common, with minimal intraoperative complications. Postoperative complications included wound hematoma, pneumonia, and dysphagia. The study concludes that multilevel corpectomies for cervical spinal osteomyelitis are safe and effective, especially with multimodal navigation utilizing intraoperative imaging and augmented reality.	The study primarily focuses on the management of subaxial cervical spine spondylodiscitis, particularly regarding ≥three-level cervical corpectomies. While the study does not specifically emphasize augmented reality (AR), it does mention the use of multimodal navigation, which includes AR, during the surgical procedure. The authors highlight the potential benefits of merging intraoperative imaging acquisition, navigation, and augmented reality for guiding implant positioning in complex anatomies and assessing optimal surgical outcomes.
*Doughty et al. (2022)* [52]	The systematic review evaluates challenges in using optical see-through head-mounted displays (OST-HMDs) for AR-assisted surgery. Fifty-seven articles from January 2021 to March 2022 were categorized based on AR navigation components. CT scans and surface-rendered models were commonly used preoperatively. Microsoft HoloLens was the primary OST-HMD, with emphasis on orthopedic and maxillofacial surgeries. Despite promising accuracy, human and technical challenges persist that need to be addressed before widespread adoption.	The systematic review investigates challenges in utilizing optical see-through head-mounted displays (OST-HMDs) for augmented reality (AR)-assisted surgery. It highlights the prevalence of Microsoft HoloLens devices and common preoperative input data sources like computed tomography (CT) scans. Virtual content is often directly superimposed onto target sites, but challenges related to perception and interaction need addressing before widespread AR adoption in surgical navigation.
*Rodriguez Peñaranda et al. (2023)* [53]	The review investigates the role of artificial intelligence (AI) in kidney cancer surgical training, utilizing advanced imaging techniques. Following PRISMA 2020 criteria, PubMed and SCOPUS databases were searched, yielding 14 eligible studies. AI applications include analyzing surgical workflows, annotating instruments, and 3D reconstruction. AI enhances surgical skill appraisal and offers benefits in intraoperative guidance and preoperative preparation. Challenges remain in regard to real-time tracking and registration. While AI shows promise in advancing surgical training with unbiased evaluations and personalized feedback, concerns regarding metric measurement, ethics, and data privacy must be addressed for full integration.	The review discusses the potential role of artificial intelligence (AI) in kidney cancer surgical training, with a focus on advanced imaging techniques. While the review primarily emphasizes AI applications, it briefly mentions augmented reality (AR) as part of the technology landscape that can enhance training. Specifically, AR could potentially contribute to surgical skill appraisal and provide benefits in terms of intraoperative guidance and preoperative preparation. However, the review does not extensively explore AR’s role compared to AI.
*Colombo et al. (2022)* [54]	The systematic review focuses on 3D segmentation and visualization techniques for brain arteriovenous malformations (bAVMs). Thirty-three studies were included, with MRI, DSA, and CT as primary imaging modalities. Automatic segmentation was predominant (61%), with a median time of 10 min. Semiautomatic and manual segmentation methods were also used. Most studies utilized screens for visualization, while only one utilized a heads-up display (HUD). Integration with mixed reality was found in four studies. The review underscores the absence of a gold standard and highlights the growing trend toward machine learning-based segmentation algorithms, particularly unsupervised fuzzy-based algorithms, suggesting ongoing efforts for improvement and innovation of visualization tools.	The systematic review primarily focuses on 3D segmentation and visualization techniques for brain arteriovenous malformations (bAVMs), without specific emphasis on augmented reality (AR). While the review discusses various visualization methods, including integration with mixed reality in four studies, it does not extensively explore AR’s role compared to other techniques. Therefore, AR’s specific application in the context of bAVM visualization is not a central focus of the review.
*Checcucci et al. (2020)* [55]	The systematic review assesses the impact of 3D printed and virtual imaging on preoperative and intraoperative aspects of robotic nephron-sparing surgery (NSS) for kidney cancer. Ten articles meeting the inclusion criteria were reviewed, with an overall ‘intermediate’ quality score and a moderate/high risk of bias across the studies. Specifically, 3D-printed models were deemed more useful for preoperative simulations and patient counseling, enhancing the understanding of anatomical structures and procedures. However, cost and material quality remain limitations. Virtual imaging in a mixed reality environment improved preoperative planning, while intraoperatively, augmented reality techniques allowed for overlaying 3D models onto real anatomy. Despite being a developing technology, virtual imaging shows promise, with potential applications expanding over time.	The systematic review primarily focuses on the impact of 3D printed and virtual imaging technologies on robotic nephron-sparing surgery (NSS) for kidney cancer, without specific emphasis on augmented reality (AR). While the review discusses the use of 3D virtual models in a mixed reality environment for preoperative planning and mentions augmented reality procedures intraoperatively, it does not extensively explore AR’s role compared to other techniques. Therefore, AR’s specific application in the context of NSS for kidney cancer is not a central focus of the review.
*Unberath et al. (2021)* [56]	The manuscript discusses the potential of image-based navigation, particularly in minimally invasive surgery, emphasizing its role in enhancing reproducibility, safety, and precision. It highlights the importance of 2D/3D registration in spatial relationship estimation between preoperative 3D structures and intraoperative 2D images, such as X-ray fluoroscopy or endoscopy. While traditional analytical solutions face challenges, the emergence of machine learning-based approaches offers promise in addressing these issues by approximating functional mapping. The review provides insights into recent advancements in machine learning’s impact on 2D/3D registration and outlines pressing needs, open problems, and potential future directions in the field.	The text primarily focuses on image-based navigation and the impact of machine learning on 2D/3D registration in the context of minimally invasive surgery. Augmented reality is cited in relation to its role at the intersection with image-based navigation, as AR technologies can enhance surgical visualization by overlaying digital information onto the surgeon’s view of the real-world surgical environment. While not directly focused on AR, in this study it is remarked that AR could potentially play a role in the broader discussion on advancing surgical workflows and mixed reality environments mentioned in the text.

**Table 2:** The theme addressed by each one of the systematic reviews.

Review Study	Theme
*Sun et al. (2023)* [41]	Revolutionary potential of VR and AR in hip surgery, emphasizing preoperative simulation, intraoperative navigation, and postoperative rehabilitation.
*Kanschik et al. (2023)* [42]	Potential of VR and AR in ICU for training, stress reduction, pain management, rehabilitation, and communication enhancement.
*Guha et al. (2017)* [43]	Pioneering role of AR in neurosurgery, focusing on image-guided surgery and the challenges and prospects associated with its precise application.
*Dubron et al. (2023)* [44]	Use of ER, AR, MR, and VR in preoperative planning of orbital fractures, highlighting AR’s role in improving surgical accuracy and precision.
*Seetohul et al. (2023)* [45]	Integration of AR into surgical robotic and autonomous systems, emphasizing the need for improved accuracy and advanced imaging techniques.
*Rodler et al. (2023)* [46]	Integration of new imaging technologies in robotic-assisted radical prostatectomy for prostate cancer, focusing on imaging modalities and their potential benefits.
*Chidambaram et al. (2021)* [47]	Application of AR technology in neurosurgery, particularly its potential to improve intraoperative visualization and surgical precision.
*Bosc et al. (2019)* [48]	Evaluation of AR applications in maxillofacial surgery, categorizing surgical approaches and emphasizing accuracy with minimal errors.
*Sparwasser et al. (2018)* [49]	Integration and future prospects of AR and VR in surgery, highlighting their potential to improve clinical outcomes, surgical training, and intraoperative procedures.
*Ong et al. (2021)* [50]	Use of ER (VR, AR, MR) in ophthalmology for education, diagnostics, and therapeutics, focusing on its potential benefits and the need for further research.
*Umana et al. (2023)* [51]	Application of augmented reality and neuronavigation in complex spinal treatments, emphasizing successful outcomes and challenges.
*Doughty et al. (2022)* [52]	Investigation of challenges with optical see-through head-mounted displays (OST-HMDs) for AR in surgery, focusing on system accuracy and technical difficulties.
*Rodriguez Peñaranda et al. (2023)* [53]	Role of artificial intelligence (AI) in renal cancer surgery training, particularly its application in advanced imaging for improved training and planning.
*Colombo et al. (2022)* [54]	Evaluation of 3D segmentation and visualization techniques for brain arteriovenous malformations (bAVMs), emphasizing the potential of automatic segmentation algorithms and the need for improvements.
*Checcucci et al. (2020)* [55]	Impact of 3D printing and virtual imaging on preoperative planning and intraoperative navigation in robotic nephron-sparing surgery in kidney cancer, highlighting their advantages and limitations.
*Unberath et al. (2021)* [56]	Role of image-based navigation in minimally invasive surgery, focusing on its potential to improve precision, safety, and cost effectiveness, and the role of machine learning in addressing registration challenges.

**Table 3:** Emerging themes in the application of AR in imaging.

Area	Potential	References
*Enhanced Visualization and Spatial Awareness*	AR overlays digital information onto the surgical field in real-time, enhancing spatial awareness and allowing for precise navigation of anatomical structures. Surgeons can visualize critical structures in a 3D space, improving surgical interventions.	[41,43,45,47,49,53]
*Preoperative Planning and Simulation*	AR integrates patient-specific imaging data into a 3D virtual environment for comprehensive preoperative planning and simulation. Surgeons can rehearse procedures, reducing risks and improving outcomes.	[41,44,49,54,55,56]
*Intraoperative Navigation and Guidance*	AR-based navigation systems provide real-time guidance to surgeons, enhancing precision and efficiency during procedures. Surgeons can accurately locate target structures and optimize instrument positioning.	[41,43,45,47,49,53]
*Education and Training*	AR offers innovative solutions for surgical education and training, enabling trainees to practice complex procedures in realistic scenarios. Immediate feedback enhances skill development and proficiency.	[41,42,44,49,50,55]
*Patient Engagement and Communication*	AR enhances patient engagement by providing interactive visualizations of medical conditions and treatment plans. Surgeons can educate patients and foster shared decision-making, improving satisfaction and adherence to treatment.	[42,49,55]
*Personalized Medicine and Surgical Precision*	AR enables personalized surgical interventions based on patient-specific data and anatomical models. Surgeons can tailor treatments for optimal outcomes and enhance precision with real-time feedback.	[41,44,49,54,55,56]
*Research and Innovation*	AR facilitates research on surgical techniques and outcomes assessments. It accelerates advancements in medical device development and the exploration of innovative solutions.	[41,45,53,56]

*AR: augmented reality.*

**Table 4:** Areas of AR integration of with other technologies in imaging applications.

Area	Description	Integrated Technologies	Studies
*Hip-related Surgery*	VAR technologies enhance precision and safety. Integration with robotics optimizes workflows, positioning, and implants.	Robotics, AI, VAR	“Virtual Reality and Augmented Reality in Hip-Related Surgery” (Reference [41])
*Intensive Care Medicine*	VAR with AI revolutionizes training and patient care. AI analyzes data, while VAR provides guidance.	Robotics, AI, VAR	“Virtual Reality and Augmented Reality in Intensive Care Medicine” (Reference [42])
*Neurosurgery*	Robotics and AI with VAR improve navigation and visualization, enhancing surgical outcomes.	Robotics, AI, VAR	“Augmented Reality in Neurosurgery” (Reference [43])
*Orbital Fractures*	Integration facilitates preoperative planning and guidance. AI analyzes data, VAR aids visualization.	Robotics, AI, VAR	“Extended Reality in Preoperative Planning for Orbital Fractures” (Reference [44])
*Renal Cell Carcinoma*	VAR with robotics and AI offer personalized treatment and training. AI customizes approaches, VAR aids visualization.	Robotics, AI, VAR	“Artificial Intelligence in Kidney Cancer Surgery” (Reference [53])

AI: artificial intelligence; VAR: virtual and augmented reality.

**Table 5:** Key suggestions for broader investigation in specific areas.

Area of Improvement	Specific Key Suggestions for Broader Investigation	References
*Preoperative Planning and Simulation*	1. Validate accuracy and clinical benefits of AR-assisted preoperative planning through rigorous clinical studies.	[44,51]
	2. Investigate AR applications in complex procedures, like orbital fractures and spinal surgeries, to assess effectiveness.	[44,51]
*Intraoperative Navigation and Guidance*	1. Address challenges, such as those related to accurate brain segmentation and hardware limitations, to scale AR use in neurosurgery.	[43,47,52]
	2. Conduct further research to enhance AR-based navigation systems for improved surgical precision and efficiency.	[41,43,45,47,49,53]
*Education and Training*	1. Evaluate and validate the efficacy of AR-driven educational tools through rigorous clinical studies.	[42,50]
	2. Integrate AR-based training into existing curricula and assess its impact on skill development and patient outcomes.	[41,42,44,49,50,55]
*Personalized Medicine and Surgical Precision*	1. Continue research and development efforts to improve technical accuracy of AR systems.	[41,44,49,54,55,56]
	2. Validate the clinical benefits of AR in personalized medicine through longitudinal studies and real-world applications.	[41,44,49,54,55,56]
*Overall Integration of AR in Surgery*	1. Foster collaborative efforts among clinicians, engineers, and researchers to address the technical challenges and validate the clinical benefits.	[41,45,53,56]
	2. Ensure seamless integration of AR technology into surgical practice by prioritizing interoperability and user-friendly interfaces.	[41,45,53,56]

**Table 6:** Common/transversal areas where the need for deeper exploration arises.

Areas Requiring Broader Investigation	References
*Integration of VR, AR, and robotics in terms of synergy*	[41,42,43,44,53]
*Clinical outcomes and comparative studies*	[41,42,43,44,45,46,47,48,49,50,51,52,53,54,55,56]
*Optimization of surgical workflows*	[41,42,43,44,45,46,47,48,49,50,51,52,53,54,55,56]
*Technological challenges and solutions*	[41,42,43,49,50,51,52,56]

*AR: augmented reality; VR: virtual reality.*

## Data Availability

No new data were created or analyzed in this study. Data sharing is not applicable to this article.

## Supplementary Materials

The following supporting information can be downloaded at: https://www.mdpi.com/article/10.3390/diagnostics14131333/s1. Section S1: Excerpts from the analyzed systematic review studies. Refs. [41,42,43,44,45,46,47,48,49,50,51,52,53,54,55,56] are cited in Supplementary Materials.
