# Supplementary material for: Exploring Augmented Reality Integration in Diagnostic Imaging: Myth or Reality?
Source: Diagnostics (Basel). 2024 Jun 23;14(13):1333. doi: 10.3390/diagnostics14131333 (PMC11240696; doi:10.3390/diagnostics14131333)
Supplement: Supplementary file 1 [file diagnostics-14-01333-s001.zip › diagnostics-3034693-supplementary.pdf]

SUPPLEMENTARY MATERIAL

S.1 Excerpts from the analyzed systematic review studies

To supplement our overview, we provide a more detailed summary of the individual systematic reviews after identifying the topics and focal points of the studies.

The systematic review by Sun et al [41] on VR and AR technology in hip surgery highlights its revolutionary potential to improve preoperative simulation, intraoperative navigation and postoperative rehabilitation. In addition, this article explores the possible future applications of AR in the operating room and discusses the promising prospects of VR and AR technologies in postoperative rehabilitation after hip surgery. The analysis of 40 studies shows that VR and AR can reduce complications, improve surgical success and contribute to safer, more precise surgeries. However, it is emphasized that further comparative studies on clinical outcomes and cost-effectiveness are needed.

Kanschik et al [42] conducted a systematic review to assess the applications of VR and AR in the intensive care unit (ICU) and found that despite the growing interest, there is a lack of comprehensive studies. The review, which included 59 studies, found that VR and AR are useful for training ICU staff in complex procedures and helping patients by reducing stress and pain and improving rehabilitation and communication with relatives. The conclusion highlights the potential of VR and AR to significantly improve the care of both medical staff and patients, with an expected increase in their application in healthcare.

Guha et al [43] investigated the pioneering role of neurosurgery in the use of AR for image-guided surgery and examined the historical development and current applications. A total of 33 articles were analyzed and included in this work. Despite challenges such as registration errors, problems with depth perception, temporal asynchrony between visual and tactile feedback, and the risk of surgeon inattention, the precise overlay of three-dimensional data on the surgical field holds great promise. The review suggests that ongoing improvements in imaging, registration accuracy, display technology and robotic integration could significantly increase the utility of AR in neurosurgical procedures.

Dubron et al [44] conducted a systematic review of the use of extended reality (ER), AR, mixed reality (MR) and VR technologies in the preoperative planning of orbital fractures. This review, based on an extensive search in major databases, highlights the ability of AR to improve surgical accuracy and precision, particularly in incision making and identification of anatomical structures. AR improves the accuracy and precision of surgical incisions and enables better identification of deep anatomical tissues in real time by improving imaging. This advance in imaging also supports precise orientation and fixation of the orbital reconstruction plate and patient-specific implants for fractured orbital walls. VR has been highlighted for its educational benefits, as it provides better visualization of craniofacial trauma compared to traditional methods. Despite these advantages, a technical accuracy margin of 2-3 mm must be taken into account for AR applications.

Seetohul et al [45] conducted a systematic review on the integration of AR into surgical robotic and autonomous systems, emphasizing the need for improved end-effector dexterity and access to minimally invasive surgery. The article explores the integration of AR with control features such as haptic

feedback to improve surgical accuracy, especially in challenging areas such as tool placement and depth perception in 2D imaging. It evaluates surgical robots according to their hardware and computer vision capabilities and emphasizes the importance of advanced imaging techniques and deep learning algorithms in overcoming obstacles in surgery. By addressing the limitations of current optimization algorithms and proposing solutions for better collision detection and image reconstruction, the review highlights the crucial role of imaging in advancing AR technology for surgical precision and safety.

Rodler et al [46] investigated the integration of new imaging technologies in robotic-assisted radical prostatectomy (RARP) for prostate cancer. They conducted a search in PubMed, Scopus and Web of Science and identified 46 relevant studies. These studies were categorized as imaging of the primary tumor, intraoperative detection of lymph nodes, and surgeon training. The review demonstrates the potential of combining magnetic resonance imaging (MRI), prostate-specific membrane antigen positron emission tomography (PSMA-PET CT) and other imaging modalities with RARP. Although the feasibility of these combined approaches has been demonstrated, the review also points out that prospective studies confirming their benefits for surgical outcomes are still in progress.

Chidambaram et al [47] conducted a systematic review of the application of AR technology in neurosurgery and analyzed its integration into clinical practice, particularly for intraoperative visualization. This review included 54 studies that focused on the use of AR in brain and spine surgery. The review highlights the potential of AR to outperform traditional neuronavigation systems in improving surgical precision and guidance. However, challenges such as accurate MRI brain segmentation, brain shifts, coregistration errors and hardware improvements remain. Future prospects include combining AR with artificial intelligence and multimodal imaging to further improve neurosurgical outcomes.

Bosc et al [48] examined AR applications in maxillofacial surgery. In this review, publications from three medical databases were analyzed and 13 studies with sufficient data for a comparative analysis were identified. Five studies emphasized a 'hands-free and heads-up' approach using smart glasses or headsets with tracking and highlighted the accuracy of AR with minimal errors below 1mm. The review categorizes surgical AR surgical applications into four types: Type I involves heads-up guided surgery, which can involve either tracking (Ia) or not (Ib); Type II uses a semi-transparent screen for guidance; Type III digitally projecting images directly onto the patient; and Type IV employs the transfer of digital data to a monitor for surgical guidance.

Sparwasser et al [49] investigated the integration and future prospects of AR and VR in surgery and emphasized their potential to improve clinical outcomes and surgical training. A systematic literature search considered 45 articles and their analysis revealed that there is a growing body of research where AR and VR applications are already finding their way into routine surgical practice, including intraoperative imaging and telementoring. Despite the enthusiasm and significant investment from technology companies, the real benefits in terms of cost savings, reducing complications and improving surgical performance have yet to be demonstrated. The next important developments in AR for surgery include improving intraoperative procedures with AR-based radiological imaging overlays, expanding remote guidance through telementoring, and improving surgical and anatomic training with AR and VR technologies. The authors call for rigorous clinical trials to evaluate these technologies and suggest that physicians should actively participate in this technological development to maximize the benefits for patients.

Ong et al [50] investigated the use of ER (VR, AR and MR) in ophthalmology, focusing on education, diagnostics and therapeutics. Among the 87 studies examined that demonstrated the use of ER in ophthalmology, the focus was on education, diagnostics and therapeutics. Although most of the studies were of poor quality, significant potential was found in improving surgical and ophthalmoscopic training, ocular imaging and the assessment of ocular disease. The results of heads-up surgical systems were in line with those of traditional surgery, emphasizing the need for further high-quality research to solidify the role of ER in ophthalmology. Despite these advances, the need for higher quality comparative studies is emphasized to fully establish the role of extended reality in ophthalmology.

Umana et al [51] presented a comprehensive literature review and case study of subaxial cervical spondylodiscitis, focusing particularly on cases requiring ≥three-level cervical corpectomies, and showed that it is a complex but safe and effective treatment for severe spinal infections involving multiple spinal levels. The systematic review included 28 patients and showed mostly successful outcomes with minimal complications and one notable case involving circumferential cervical fixation and four-level cervicothoracic corpectomy enhanced by augmented reality and neuronavigation. This approach shows promising results in the treatment of extensive cervical spinal osteomyelitis despite the challenges of spinal deformities and instability and emphasizes the importance of advanced surgical techniques and multimodal navigation to achieve optimal results.

Doughty et al [52] conducted a systematic review to investigate the use of optical see-through head-mounted displays (OST-HMDs) for AR in surgery and analyzed 57 relevant articles from January 2021 to March 2022. This study showed a focus on orthopedic and maxillofacial surgery, using CT scans and surface models as common preoperative data sources. While the Microsoft HoloLens has been widely used for its interface capabilities, there are still challenges with system accuracy, user perception and technical difficulties such as occlusion and interaction. Despite promising accuracy of 2-5 mm in phantom models, these issues need to be resolved for OST HMDs to gain wider acceptance in surgical navigation.

Rodriguez Peñaranda et al [53] investigated the role of artificial intelligence (AI) in renal cancer surgery training, focusing on its application in advanced imaging to improve training and planning. A total of 14 eligible studies were selected and analyzed. AI facilitates surgical training by analyzing surgical workflows, instrument labeling, tissue identification and 3D reconstruction, improving preoperative preparation and intraoperative guidance. While AI and AR promise to refine surgical training through unbiased assessments and personalized feedback, challenges with real-time tracking, privacy and ethical concerns remain, highlighting the need for further research to optimize the integration of AI into surgical imaging and training.

Colombo et al [54] evaluated the current status of 3D segmentation and visualization techniques for brain arteriovenous malformations (bAVMs) and investigated their role in the characterization and management of these complex lesions. The study summarizes the results of thirty-three studies and highlights that automatic segmentation algorithms based on machine learning are a potentially preferable method due to their efficiency and lower time requirements compared to semi-automatic and manual segmentation. Despite the widespread use of screens for 3D visualization, some studies are looking at mixed reality and heads-up displays, indicating a lack of consensus on a gold standard for bAVM visualization. This underlines the ongoing need to improve algorithms and innovative visualization tools.

Checcucci et al [55] investigated the impact of 3D printing and virtual imaging on preoperative planning and intraoperative navigation in robotic nephron-sparing surgery in kidney cancer. From the analysis of ten included articles, this study found that 3D-printed models improve preoperative simulations and patient counseling by improving the understanding of anatomical structures. However, the main limitations of 3D printing include the cost and quality of materials. Conversely, VR and MR environments offer significant advantages in preoperative planning and enable AR-assisted procedures during surgery, representing a rapidly developing field with growing application possibilities.

Unberath et al [56] investigated the role of image-based navigation in minimally invasive surgery and emphasized its potential to make highly precise, safe and cost-effective surgery more accessible by integrating it into current workflows and facilitating MR, autonomous and robot-assisted surgery. A major focus is on 2D/3D registration, which is crucial for mapping 3D structures onto intraoperative 2D images and faces challenges such as optimization difficulties and limited performance on certain views. Machine learning represents a promising solution as it offers new ways to address these challenges by approximating complex functional mappings without specifying them directly. This systematic review highlights the need for further research to address current problems and explore the potential of machine learning in improving 2D/3D registration techniques.
